# Fingolimod retains cytolytic T cells and limits T follicular helper cell infection in lymphoid sites of SIV persistence

**DOI:** 10.1371/journal.ppat.1008081

**Published:** 2019-10-18

**Authors:** Maria Pino, Sara Paganini, Claire Deleage, Kartika Padhan, Justin L. Harper, Colin T. King, Luca Micci, Barbara Cervasi, Joseph C. Mudd, Kiran P. Gill, Sherrie M. Jean, Kirk Easley, Guido Silvestri, Jacob D. Estes, Constantinos Petrovas, Michael M. Lederman, Mirko Paiardini

**Affiliations:** 1 Division of Microbiology and Immunology, Yerkes National Primate Research Center, Emory University, Atlanta, Georgia, United States of America; 2 AIDS and Cancer Virus Program, Frederick National Laboratory for Cancer Research, Leidos Biomedical Research, Inc., Frederick, Maryland, United States of America; 3 Tissue Analysis Core, Immunology Laboratory, Vaccine Research Center, NIAID, NIH, Bethesda, Maryland, United States of America; 4 Flow Cytometry Core, Emory Vaccine Center, Emory University, Atlanta, Georgia, United States of America; 5 Center for AIDS Research, Department of Medicine, Case Western Reserve University and University Hospitals, Cleveland Medical Center, Cleveland, Ohio, United States of America; 6 Division of Animal Resources, Yerkes National Primate Research Center, Emory University, Atlanta, Georgia, United States of America; 7 Department of Biostatistics and Bioinformatics, Rollins School of Public Health, Emory University, Atlanta, Georgia, United States of America; 8 Department of Pathology and Laboratory Medicine, Emory University School of Medicine, Atlanta, Georgia, United States of America; 9 Vaccine and Gene Therapy Institute at Oregon Health Science Center, Portland, Oregon, United States of America; Imperial College London, UNITED KINGDOM

## Abstract

Lymph nodes (LN) and their resident T follicular helper CD4+ T cells (Tfh) are a critical site for HIV replication and persistence. Therefore, optimizing antiviral activity in lymphoid tissues will be needed to reduce or eliminate the HIV reservoir. In this study, we retained effector immune cells in LN of cART-suppressed, SIV-infected rhesus macaques by treatment with the lysophospholipid sphingosine-1 phosphate receptor modulator FTY720 (fingolimod). FTY720 was remarkably effective in reducing circulating CD4+ and CD8+ T cells, including those with cytolytic potential, and in increasing the number of these T cells retained in LN, as determined directly *in situ* by histocytometry and immunohistochemistry. The FTY720-induced inhibition of T cell egress from LN resulted in a measurable decrease of SIV-DNA content in blood as well as in LN Tfh cells in most treated animals. In conclusion, FTY720 administration has the potential to limit viral persistence, including in the critical Tfh cellular reservoir. These findings provide rationale for strategies designed to retain antiviral T cells in lymphoid tissues to target HIV remission.

## Author summary

FTY720 (fingolimod), a drug approved by the FDA for treatment of multiple sclerosis, blocks the egress of lymphocytes from the lymph node (LN). To determine whether FTY720 retention activity could improve cytolytic responses in the LN and affect SIV persistence, we studied for the first time tolerability and biological activity of two doses of FTY720 in cART-suppressed, SIV-infected rhesus macaques. FTY720 was remarkably effective in reducing circulating CD4+ and CD8+ T cells, including those with cytolytic potential, and in increasing the number of cytolytic T cells in LN. FTY720 administration reduced SIV-DNA content in blood as well as in LN Tfh cells in most of the animals. These results suggest that FTY720 limits viral persistence, including Tfh cellular reservoir, by increasing the number of cytolytic cells in the LN, critical site for HIV/SIV replication and persistence.

## Introduction

One of the greatest therapeutic challenges in HIV research and care is that of cure. The major barrier to cure is the reservoir of latently infected cells, mostly resting memory CD4+ T cells, containing replication competent proviruses that persist in spite of prolonged combination antiretroviral therapy (cART) [1–5]. When cART is interrupted, HIV levels in plasma typically rebound in the vast majority of individuals [6, 7]. Furthermore, cART does not completely eliminate the persistent low level inflammation [8] that is linked to an increased risk of neurologic, malignant and cardiovascular disease [9–11]. Even when started during the early phase of HIV infection, cART is rarely sufficient to permit even functional control of HIV replication and there is a need to design innovative therapies able to eliminate or at least limit HIV persistence particularly among persons with chronic infection who represent the vast majority of HIV-infected individuals. It is becoming increasingly clear that any strategy that attempts HIV eradication will need to address the persistence of virus in tissue sites such as secondary lymphoid organs in cART-suppressed, HIV-infected individuals [12] and SIV-infected rhesus macaques (RMs;[13]). Several factors play a key role in the persistence of HIV in lymphoid organs: these sites are rapidly infected in early infection [14], maintain residual level of activation/inflammation that may potentiate infection of susceptible cells to contribute to the latent reservoir [15], contain a network of follicular dendritic cells in which virions may persist durably [16], and may show suboptimal penetration of otherwise effective antiretroviral drugs [17]. Supporting the critical role of LN in HIV persistence, recent studies showed that (i) B cell follicles in the LN constitute sanctuaries for persistent SIV replication in RM elite controllers [18], (ii) LN follicular helper T cells (Tfh) represent the major CD4 T cell compartment for HIV replication in viremic individuals [19, 20]; (iii) LN CD4+ T cells that express programmed cell death 1 (PD-1) are the major sites of HIV transcription in cART-treated HIV-infected individuals [12]; and (iv) PD-1+ Tfh cells and CTLA-4+PD-1- Treg cells harboring replication competent virus persist in the LN B cell zone and T cell zone, respectively, of cART-treated, SIV-infected RMs [13].

For these reasons, maintenance of an effective antiviral immune response seems particularly critical at lymphoid sites. Cytolytic effector T cells are however typically excluded from LN, moving across a concentration gradient of the lysophospholipid sphingosine-1 phosphate (S1P) to exit the nodes and enter circulation [21]. Furthermore, it is unclear how many of the LN resident CD8+ T cells are able to upregulate the germinal center homing molecule CXCR5 and to enter follicles, where they can encounter Tfh cells. While previous studies supported a model in which SIV-specific CD8+ T cells were largely excluded by LN follicles [22], other recent works suggest that at least a fraction of CD8+ T cells can upregulate CXCR5 and homing into the follicles, in particular after activation by virus or by immunization [23, 24].

S1P binds a family of five G protein-coupled S1P receptors (S1PR_1-5_), which are expressed in multiple cell types, allowing their activation, triggering a signal that targets different pathways involved in cell survival, proliferation, and, importantly, egress from the LN [25–28]. Lymphoid hyperplasia and sequestration of immune cells in lymph nodes was characteristic of untreated HIV infection [29] and decreased S1P function has been demonstrated among LN T cells from viremic HIV-infected individuals [30]. These defects were largely corrected with antiretroviral therapy that is associated with the first phase rapid release of lymphocytes from lymph nodes into circulation [31–33]. Thus, natural host immune homeostatic mechanisms that exclude cytolytic effector cells from lymphoid tissues may limit the ability to target these sites of HIV persistence in cART-suppressed HIV-1-infected individuals.

Fingolimod (FTY720; 2-amino-2-[2-(4-octylphenyl)ethyl]propane-1,3-diol), derived from a metabolite (myriocin) of the fungus *Isaria sinclairii*, has been shown to act as a S1PR modulator by binding and blocking the interaction of S1P with four of its receptors (S1PR1, S1PR3, S1PR4 and S1PR5) [34], thus inducing a profound peripheral lymphopenia [28, 35–40]. FTY720 is effective and approved by the FDA for the treatment of multiple sclerosis [41–43]. This clinical effect is thought to be related in part to the sequestration of activated T cells in lymphoid tissue and prevention of their migration to the central nervous system [44–47]. In this study, we treated ten cART-suppressed SIV-infected RMs with two doses of FTY720, with the aim of assessing its tolerability and evaluating its ability to retain T cells in lymph nodes and affect indices of SIV persistence.

## Results

### FTY720 administration is well-tolerated and reduces levels of circulating T cells in cART-treated, SIV-infected RMs

**Fig 1:**
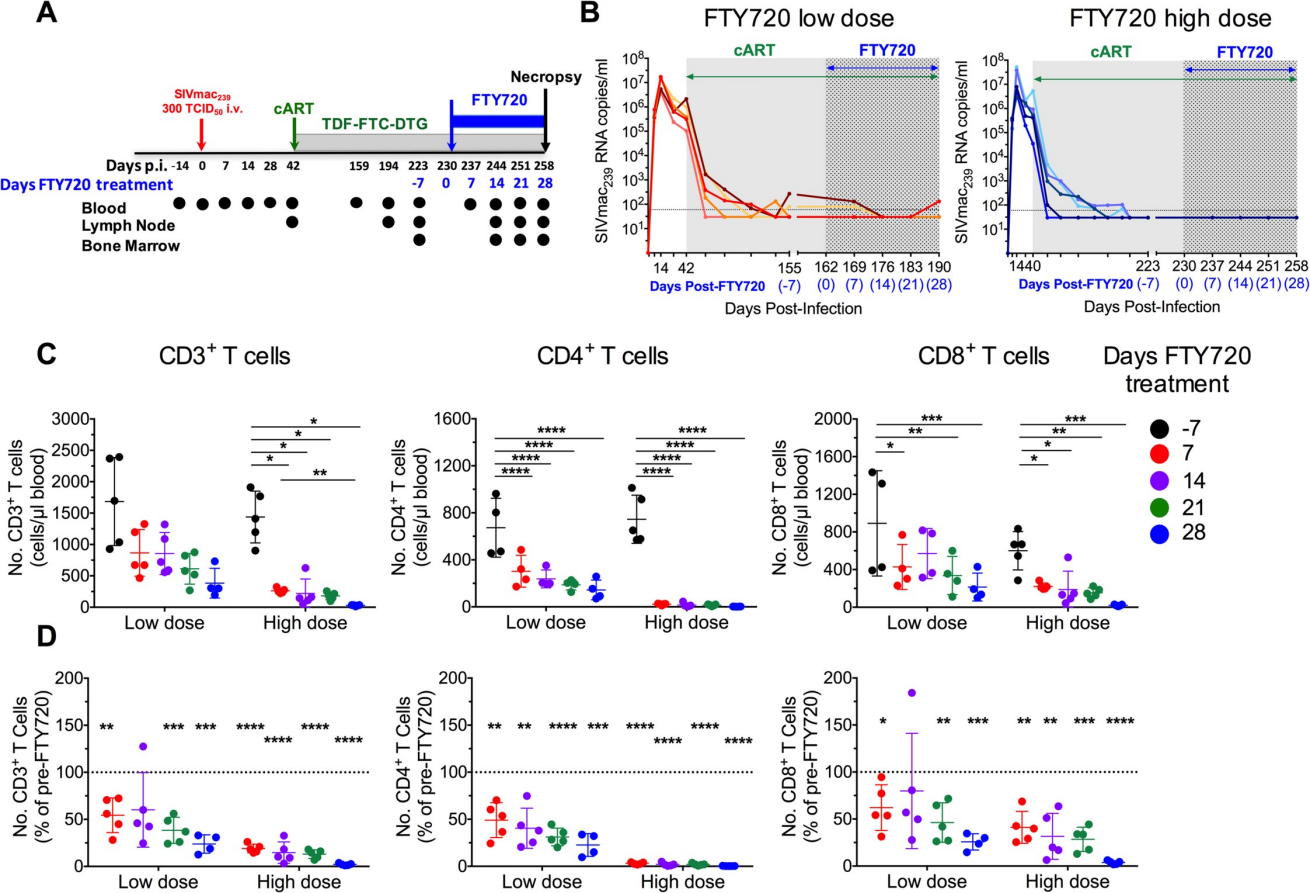
FTY720 reduces levels of circulating T cells in cART-treated, SIV-infected RMs. **(A)** Schematic of the study design. **(B)** Plasma SIV_mac239_ RNA levels expressed as copies/ml (LOD, 60 copies/ml, dashed line) are shown for each individual animal from low dose group (left panel) and high dose group (right panel). cART and FTY720 treatments are indicated in green and blue arrows, respectively. **(C)** Absolute numbers (cells/μl) of circulating CD3+, CD4+, and CD8+ T cells at day -7 (pre-FTY720), and days 7, 14, 21, and 28 of FTY720 treatment. In **(D),** the numbers of circulating CD3+, CD4+, and CD8+ T cells at days 7, 14, 21, and 28 of FTY720 treatment are presented as proportion of their baseline levels. cART, combination ART. Data are presented as the mean ± SD. Statistical differences in (**C, D**) were assessed with a two-way ANOVA or a one sample t-test. *P ≤ 0.05, **P ≤ 0.01, ***P ≤ 0.001, ****P ≤ 0.0001.

Ten RMs were infected intravenously (i.v.) with 300 TCID_50_ SIV_mac239_ and, starting from day 42 post infection (p.i.), treated with a potent, combined ART regimen (tenofovir, 5.1 mg/Kg per day; emtricitabine, 40 mg/Kg per day; and dolutegravir, 2.5 mg/Kg per day) co-formulated in a single daily subcutaneous injection (**Fig 1A**). cART was continued up to day 258 p.i., and reduced plasma viremia to undetectable level (<60 copies/mL) in all animals (**Fig 1B** and **S1 Table**; ROj10 and RUs11 in low dose group had a blip at 80 and 270 copies/mL just before FTY720 treatment initiation). Low dose group RMs (n = 5) received FTY720 at 18 μg/Kg per day and high dose group (n = 5) at 500 μg/Kg per day. The 18 μg/Kg per day dose corresponds to the 1.25 mg per day dose safely used in two clinical trials with more than 800 multiple sclerosis patients [42, 43] NCT00340834 and NCT00289978); the higher dose of 500 μg/Kg per day was chosen since doses up to 1 mg/Kg have been given to RMs in transplantation studies [48, 49]. FTY720 was administered orally once a day during the last 28 days of cART, once viremic control was achieved (day 162 p.i. for low dose group and day 230 p.i. for high dose group; **Fig 1B** and **S1 Table**). Neither dose of FTY720 was associated with significant change from baseline (pre-FTY720) in serum chemistries or hematologic indices (**S1A Fig and S2 Table**), and all 10 RMs maintained stable body-weight (**S1B Fig**). Plasma viremia remained below the limit of detection during FTY720 treatment (**Fig 1B** and **S1 Table**), in all but RLr11 (low dose group) that experienced a small blip during FTY720 treatment (130 copies/mL, d 28). Thus, all 10 RMs completed the 28 days of FTY720 treatment without complication or toxicity, supporting the tolerability and feasibility of FTY720 supplementation at doses up to 500 μg/Kg per day during cART treatment.

We next investigated the effect of FTY720 administration on lymphocyte distribution. Absolute numbers of CD3+, CD4+, and CD8+ T cells were enumerated during cART at baseline (pre-FTY720; d -7) and weekly during FTY720 treatment (d 7, 14, 21, and 28). FTY720 induced a statistically significant, dose-dependent reduction in the absolute numbers of all three cell populations. Circulating T cell numbers fell rapidly at the first reading (d 7), and dropped progressively during treatment **(Fig 1C)**. Specifically, by the last day of FTY720 administration (d 28), in high dose group animals CD3+ T cells (cells/μl) were reduced from 1437±412 to 28±10; CD4+ T cells were reduced from 745±205 to 2±1; CD8+ T cells were reduced from 600±203 to 21±8. The dose-dependent reduction in circulating lymphocyte numbers is underscored by expressing cell counts as proportional decreases from baseline (**Fig 1D**). Circulating counts of CD3+, CD4+, and CD8+ T cells at d 28 of FTY720 treatment were reduced, respectively, to an average of 23.8%, 22.6%, and 25.4% of initial values in low dose group, and to 2.1%, 0.3%, and 3.8% of initial values in high dose group. Finally, and consistent with expression of targeted forms of S1PR [50], FTY720 also reduced circulating numbers of CD3-CD20+ B cells, while the effects on CD3-HLADR-CD20-CD8+NKG2A/C+ natural killer (NK) cells were less pronounced than those of T and B cells (**S2 Fig**).

### FTY720 induces a transient increase in the frequencies of cycling T cells in circulation of cART-treated, SIV-infected RMs

**Fig 2:**
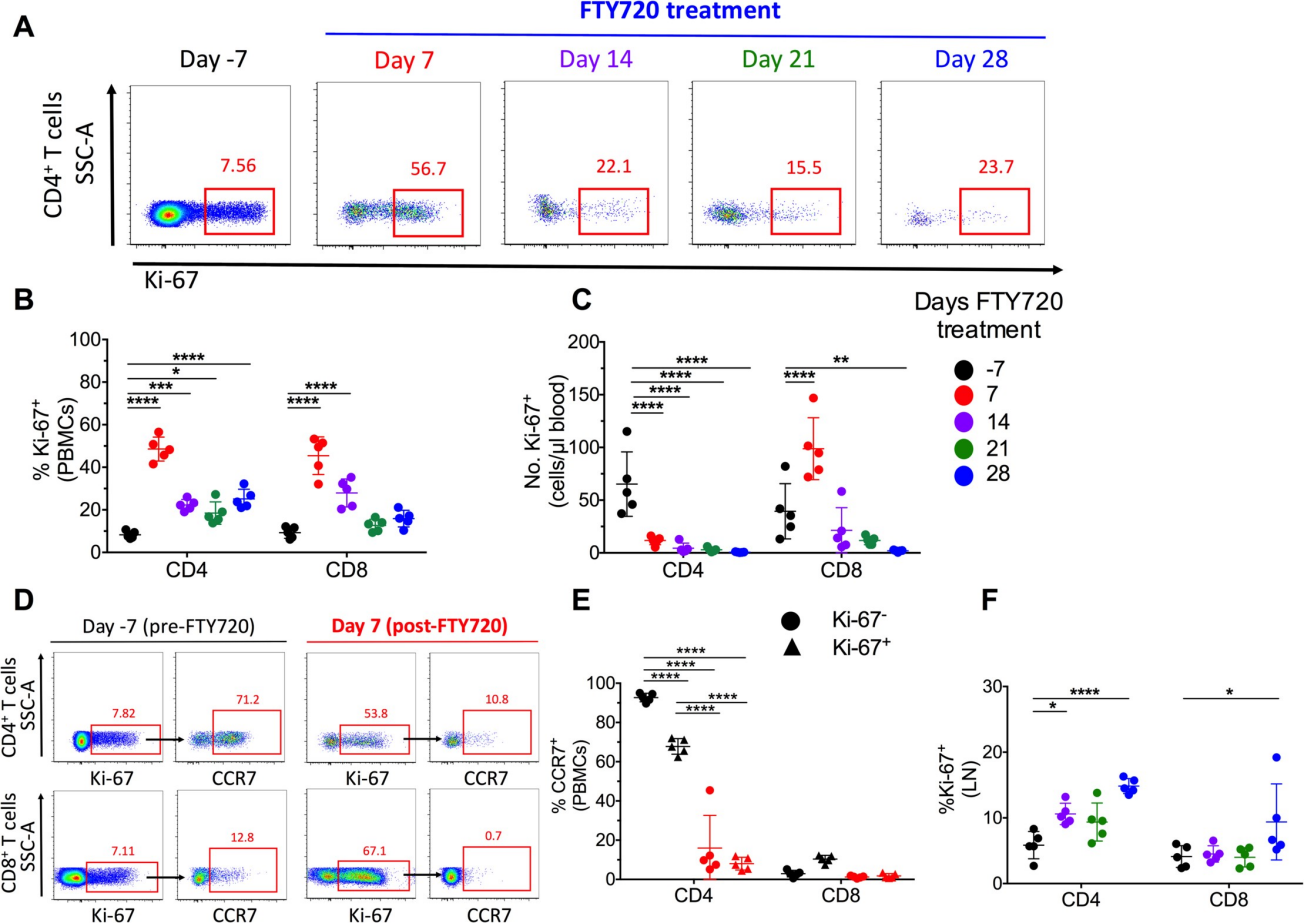
FTY720 increases the frequencies of cycling T cells in blood of cART-treated, SIV-infected RMs. **(A)** Representative Ki-67 staining on CD4+ T cells at day -7 (pre-FTY720), and days 7, 14, 21, and 28 of FTY720 treatment. **(B)** Frequency of CD4+ and CD8+ T cells expressing Ki-67 pre- and post-FTY720 treatment for high dose group in peripheral blood mononuclear cells (PBMCs). **(C)** Absolute numbers (cells/μl) of Ki-67+ CD4+ and CD8+ T cells pre- and post-FTY720 treatment for high dose group in blood. **(D)** Representative staining for Ki-67+ and CCR7+ CD4+ and CD8+ T cells pre-FTY720 and at day 7 of FTY720 treatment in blood. **(E)** Frequency of CCR7+ expression on Ki-67+ or Ki-67- blood CD4+ and CD8+ T cells pre- and at day 7 of FTY720 treatment. **(F)** Frequency of CD4+ and CD8+ T cells expressing Ki-67 in lymph node (LN) pre- and post-FTY720 treatment (high dose group). Data are presented as the mean ± SD. Statistical differences were assessed with a two-way ANOVA. *P ≤ 0.05, **P ≤ 0.01, ***P ≤ 0.001, ****P ≤ 0.0001.

To further define the effects of FTY720 on T cell immune homeostasis, we examined expression of the cell cycling marker Ki-67 in peripheral blood mononuclear cells (PBMCs). The highest dose of FTY720 induced a rapid and significant increase in the percentage of T cells expressing Ki-67 (a representative staining is shown in **Fig 2A**). Specifically, in high dose group the frequency of CD4+ and CD8+ T cells expressing Ki-67 increased from 8.2±1.7% and 9.3%±2.6% at baseline to 48.6±5.6% and 45.4±8.8% at d 7 of FTY720 treatment, respectively (**Fig 2B).** Proportions of cycling T cells then declined, although they remained significantly higher than at baseline until the end of the FTY720 treatment. Despite the significant increase in the proportion of cycling cells, and as a result of a massive depletion of circulating CD4+ T cells already at d 7 post FTY720 treatment, the absolute number of CD4+Ki-67+ T cells remained significantly lower than at baseline at all experimental points (**Fig 2C**). Yet, the absolute number of CD8+Ki-67+ T cells was significantly higher at d 7 post FTY720 than at baseline but then decreased progressively to significantly lower levels than at baseline at d 21 and 28 of treatment (**Fig 2C).** The frequencies of cycling T cells in blood increased minimally and not significantly in low dose group animals. One possible mechanism for increased proportion but decreased absolute number of circulating Ki-67+ T cells could be a reduced expression on cycling cells of CCR7, a chemokine receptor that promotes leukocyte homing to lymphoid sites. This would preferentially maintain cycling cells in blood as they could not enter lymphoid tissues. To this end, we quantified the frequency of CD4+ and CD8+ T cells that express CCR7 based on their Ki-67 status before and at d 7 post FTY720, the latter corresponding to the peak Ki-67+ T cell frequency. A representative figure of CCR7 by Ki-67 staining is shown in **Fig 2D**. At baseline, CD4+Ki-67+ T cells express CCR7 at lower frequency than CD4+Ki-67- cells (67.8% vs 92.7%; p< 0.0001); in contrast, CD8+Ki-67+ cells more frequently express CCR7 than do CD8+ Ki-67- cells (10.4% vs. 2.9%; not significant) (**Fig 2E**). The frequency of CD4+ and CD8+ T cells expressing CCR7 was very low for both Ki-67+ and Ki-67- cells at d 7 post FTY720, consistent with the proposed mechanism of action of FTY720, i.e. an active entrapment of CCR7+ T cells in lymphoid tissues (**Fig 2E**). Together, these data suggest that the increased frequency of CD4+Ki-67+ T cells in circulation following FTY720 treatment is likely related, at least in part, to a lower potential for cycling cells to home (and be retained) in lymphoid tissues. This mechanism does not seem to be important for CD8+ T cells. The fraction of cycling CD4+ T cells in LN significantly increased at all measured experimental points during FTY720 treatment as compared to baseline (**Fig 2F**), although proportions were lower than those found in blood. The fraction of CD8+Ki-67+ T cells in LN was significantly higher than baseline only at d 28 of FTY720 treatment (**Fig 2F**). Finally, since bone marrow (BM) has been emerging as a critical site for memory T cell homeostasis [51], and CD4+ T cells in the BM have been shown to be depleted following SIV infection and to contribute to the size of the replication competent reservoir [52], we investigated how FTY720 treatment impacted on BM T cells. Consistently with blood, also in BM we found a significant reduction in the levels of CD3+, CD4+, and CD8+ T cells expressed as frequency of total live lymphocytes, that was more pronounced for the high dose group (**S3A–S3C Fig**). Furthermore, FTY720 treatment also resulted in a significant frequency of CD4+Ki-67+ T cells at day 14 and 21 post FTY720 for both treatment groups; those frequencies were reduced to baseline levels at day 28 post treatment (**S3D and S3E Fig**). Thus, FTY720 administration reduced levels of blood and BM T cells and transiently increased their capacity to enter cell cycle in cART-treated, SIV-infected RMs.

### FTY720-induced reduction of circulating cells involves all T cell subsets, including those producing cytotoxic molecules

**Fig 3:**
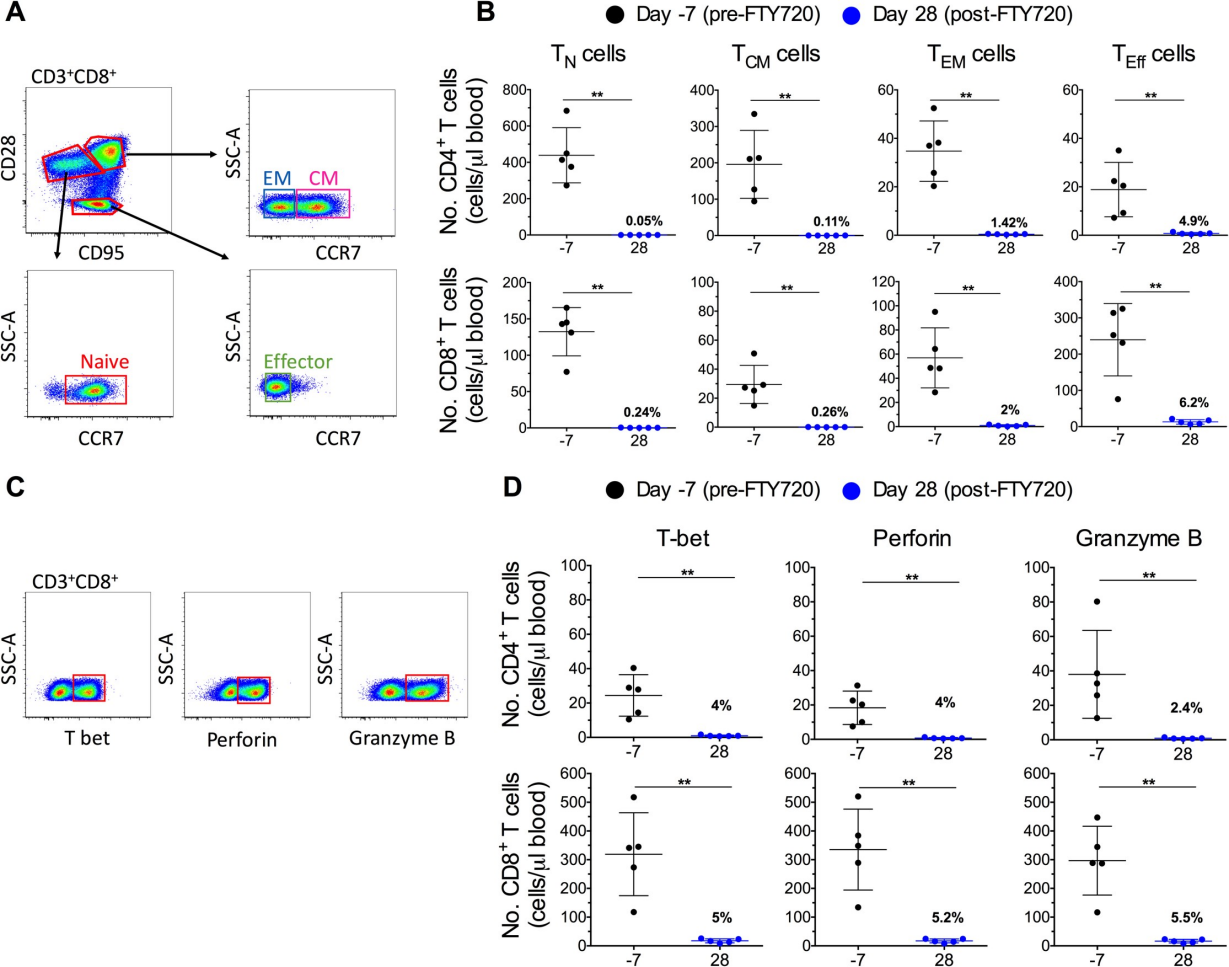
FTY720 induces a reduction of all circulating T cell subsets, including those producing cytotoxic molecules. **(A)** Representative staining of different T cell subsets including naïve (T_N_), central memory (T_CM_), effector memory (T_EM_), and effector (T_Eff_) T cells in blood. **(B)** CD4+ (top panels), and CD8+ (bottom panels) T cell subsets expressed in absolute numbers (cells/μl) at day -7 (pre-FTY720; black dots) and day 28 (post-FTY720; blue dots) for high dose group. **(C)** Representative staining of CD8+ T cells expressing cytolytic molecules: T-bet, perforin, and granzyme B in blood. **(D)** Absolute numbers (cells/μl) of CD4+ (top panels) and CD8+ (bottom panels) T cells expressing perforin, T-bet, or granzyme B in blood pre- and post-FTY720 (high dose group). Data are presented as the mean ± SD. Statistical differences were assessed with a Mann-Whitney u-test in **(B)**, and **(D)**. *P ≤ 0.05, **P ≤ 0.01, ***P ≤ 0.001, ****P ≤ 0.0001.

As expression of S1P receptors may vary according to cellular activation or maturation status, we next examined the effects of FTY720 administration on CD4+ and CD8+ T cell subset numbers, defined phenotypically as naïve (T_N_; CD28+CD95-CCR7+), central memory (T_CM_; CD28+CD95+CCR7+), effector memory (T_EM_; CD28+CD95+CCR7-) and effector (T_Eff_; CD28-CD95+CCR7-) cells. A representative staining and gating strategy for these different subsets is shown for blood CD8+ T cells (**Fig 3A)**. Absolute numbers of each T cell subset were significantly reduced from baseline at d 28 of FTY720 treatment in both low dose group (with the exception of CD8+ T_Eff_; **S4A Fig**), and high dose group (**Fig 3B**). In high dose group, absolute counts of T_N_, T_CM_, T_EM_, and T_E_ CD4+ cells after 28 days of FTY720 treatment were reduced to an average of 0.05%, 0.11%, 1.42% and 4.9% of the baseline levels, respectively (**Fig 3B**). Among CD8+ T cells, the same subsets fell to 0.24%, 0.26%, 2.0% and 6.2% of baseline levels (**Fig 3B)**. The majority of the very few remaining T cells in circulation during FTY720 treatment express a T_Eff_ phenotype, particularly among the CD8+ T cells.

Finally, we examined the changes in the absolute counts and relative frequencies of circulating T cells expressing T-bet, perforin, and granzyme B (molecules associated with antiviral function and cytolytic activity). A representative staining for these markers is shown for CD8+ T cells in **Fig 3C**. The absolute counts (cells/μl of blood) of CD8+ T cells expressing T-bet, perforin, and granzyme B were reduced in high dose group from 319±144, 335±141, and 297±120 at baseline (pre-FTY720) to only 17±7 (5.0% of their baseline value), 17±7 (5.2% of baseline), and 16±6 (5.5% of baseline) after 28 days of FTY720 treatment (**Fig 3D**). Similarly, in high dose group absolute counts (cells/μl of blood) of CD4+ T cells expressing T-bet, perforin, and granzyme B were reduced from 24±12, 18±10, and 38±26 to 1±0.4, 0.75±0.3, and 0.9±0.35 (4.0%, 4.0%, and 2.4% of baseline, respectively) after FTY720 treatment (**Fig 3D**). The absolute numbers of CD4+ and CD8+ T cells expressing T-bet, perforin, and granzyme B were reduced also with the lower dose of FTY720 treatment, although without reaching statistical significance (**S4B Fig**).

In summary, FTY720 administration induced profound, dose-dependent decreases in circulating CD4+ and CD8+ T lymphocytes including those with cytolytic potential in cART-suppressed, SIV-infected RMs.

### FTY720 increases T cell accumulation in LN

**Fig 4:**
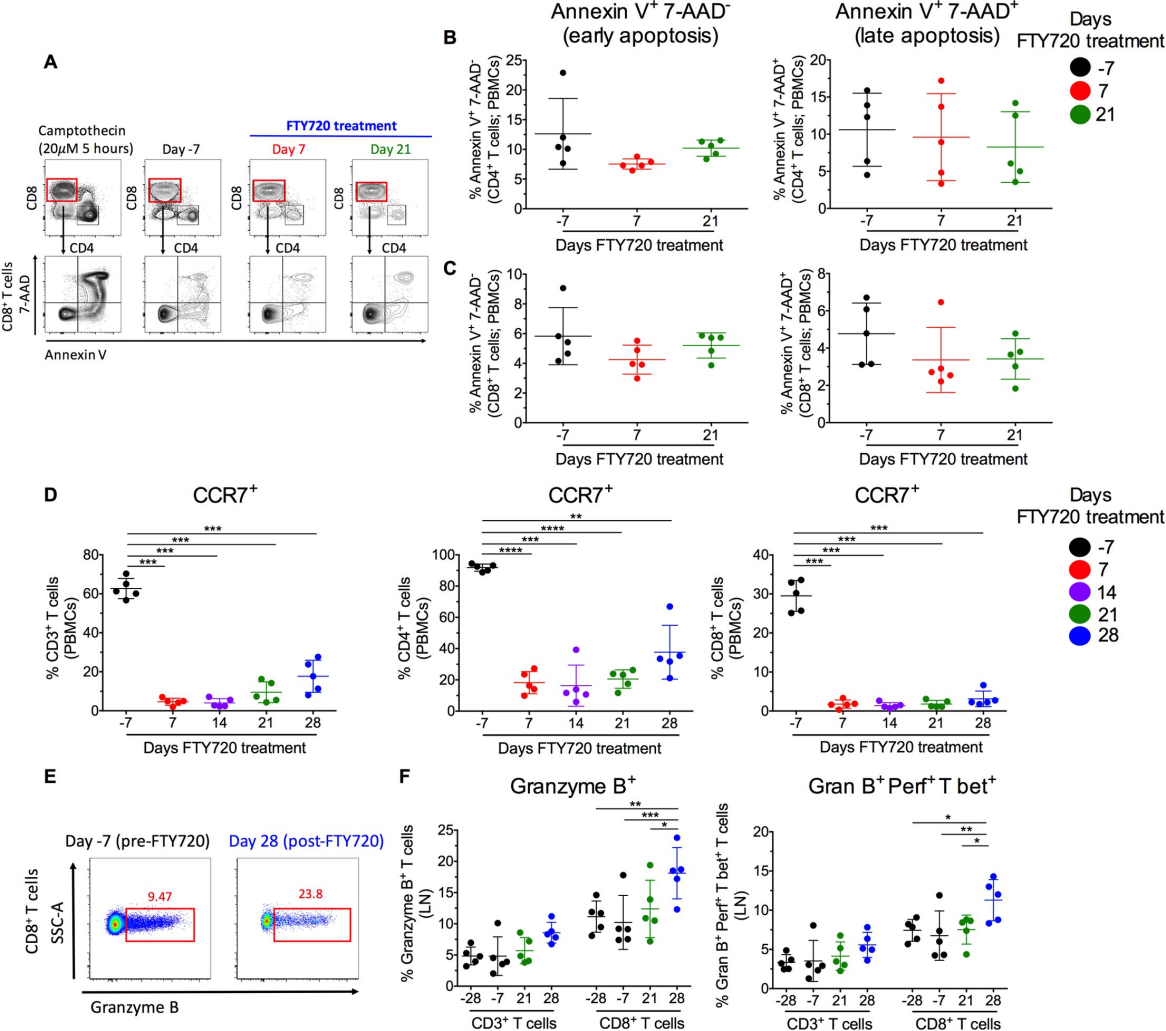
FTY720 mediated loss of circulating T cells is due to their increased homing to LN. **(A)** Representative staining of CD8+ T cells expressing 7-AAD and Annexin V in peripheral blood mononuclear cells (PBMCs) before and after FTY720 treatment. PBMCs from a healthy SIV-uninfected RM incubated for 5 hours with 20μM camptothecin, used as a positive control, are also shown. **(B,C)** Frequency of blood CD4+ and CD8+ T cells expressing Annexin V alone (early apoptosis; right panel) or Annexin V and 7-AAD (late apoptosis; left panel) at pre-, and post-FTY720 treatment in the high dose group. **(D)** Expression of the homing marker CCR7+ on CD3+ (left panel), CD4+ (middle panel), and CD8+ (right panel) T cells at pre-, and post-FTY720 treatment for high dose group in blood (PBMCs). **(E)** Representative gating strategy of CD8+ T cells expressing granzyme B+ at pre-, and post-FTY720 treatment in lymph node (LN). **(F)** Expression of granzyme B+ (left panel), and co-expression of granzyme B+, perforin+, and T-bet+ (right panel) on CD3+ and CD8+ T cells at pre-, and post-FTY720 treatment in LN. Statistical differences were assessed with a one-way ANOVA test in **(B), (C), (D)** or **(F)**. *P ≤ 0.05, **P ≤ 0.01, ***P ≤ 0.001, ****P ≤ 0.0001.

To exclude the possibility that the reduction of lymphocytes in circulation is due to cell death, we first performed experiments in which we determined by flow cytometry the frequency of circulating T cells that are in the early or late phase of apoptosis, based on the binding of Annexin V and staining with 7- Aminoactinomycin (7-AAD) (representative staining in **Fig 4A**). Despite limited to few events due to the severe loss of circulating T cells, our analyses showed that the frequency of CD4+ and CD8+ T cells with a phenotype of early (Annexin V+7-AAD-) or late (Annexin V+7AAD+) apoptosis did not increase following FTY720 administration in the high dose group (**Fig 4B and 4C**). Thus, increased apoptosis contributes minimally, if any, to the loss of T cells from blood. We next asked if the extensive loss of T cells from blood following FTY720 treatment resulted in a measurable increase in LN T cells. We quantified the frequency of T cells in blood expressing the chemokine receptor CCR7 that promotes leukocyte homing to lymphoid sites. In high dose group, the percentages of CD3+, CD4+, and CD8+ T cells expressing CCR7 were reduced from 62.7±5.2, 91.8±2.3, and 29.5±3.9 pre-FTY720 to 4.6±1.8, 18.2±7.1, and 1.8±1.1 at d 7 post-FTY720, respectively, and remained consistently lower than baseline until the end of treatment (**Fig 4D**). Thus during FTY720 treatment, peripheral blood is profoundly depleted of T cells capable of trafficking to LN, suggesting that cells with that capacity were retained in lymphoid tissues. Furthermore, we determined the frequency of T, NK, and B cells in the LN before FTY720 (baseline) and at multiple experimental points during FTY720 treatment by flow cytometry. The frequency of both CD4+ and CD8+ T cells remained stable, likely due to FTY720 activity in blocking the egress of multiple lymphocyte subsets and the recognition that quantifying absolute numbers of LN cells in suspension (as performed in blood) is not possible (**S5A and S5B Fig**). The frequency of NK (**S5C Fig**) and B (**S5D Fig**) cells also remain relatively stable during the treatment, but were significantly reduced in the high dose group at the latest day of treatment. This finding suggests a higher retention of T cells as compared to NK and B cells, consistent with the more pronounced loss of T cells than NK and B cells in circulation (**Figs 1C** and **S2B**). Next, we analyzed the levels of T cells expressing granzyme B, perforin and T-bet in LN before and after FTY720 treatment. The frequency of CD3+ and CD8+ T cells expressing granzyme B (a representative granzyme B staining is shown for CD8+ T cells in **Fig 4E**) or triple positive for granzyme B, perforin, and T-bet, although stable (or decreasing) during the two baseline measuraments, were progressively and significantly (d 28 as compared to baseline) increased during FTY720 treatment (**Fig 4F**).

**Fig 5:**
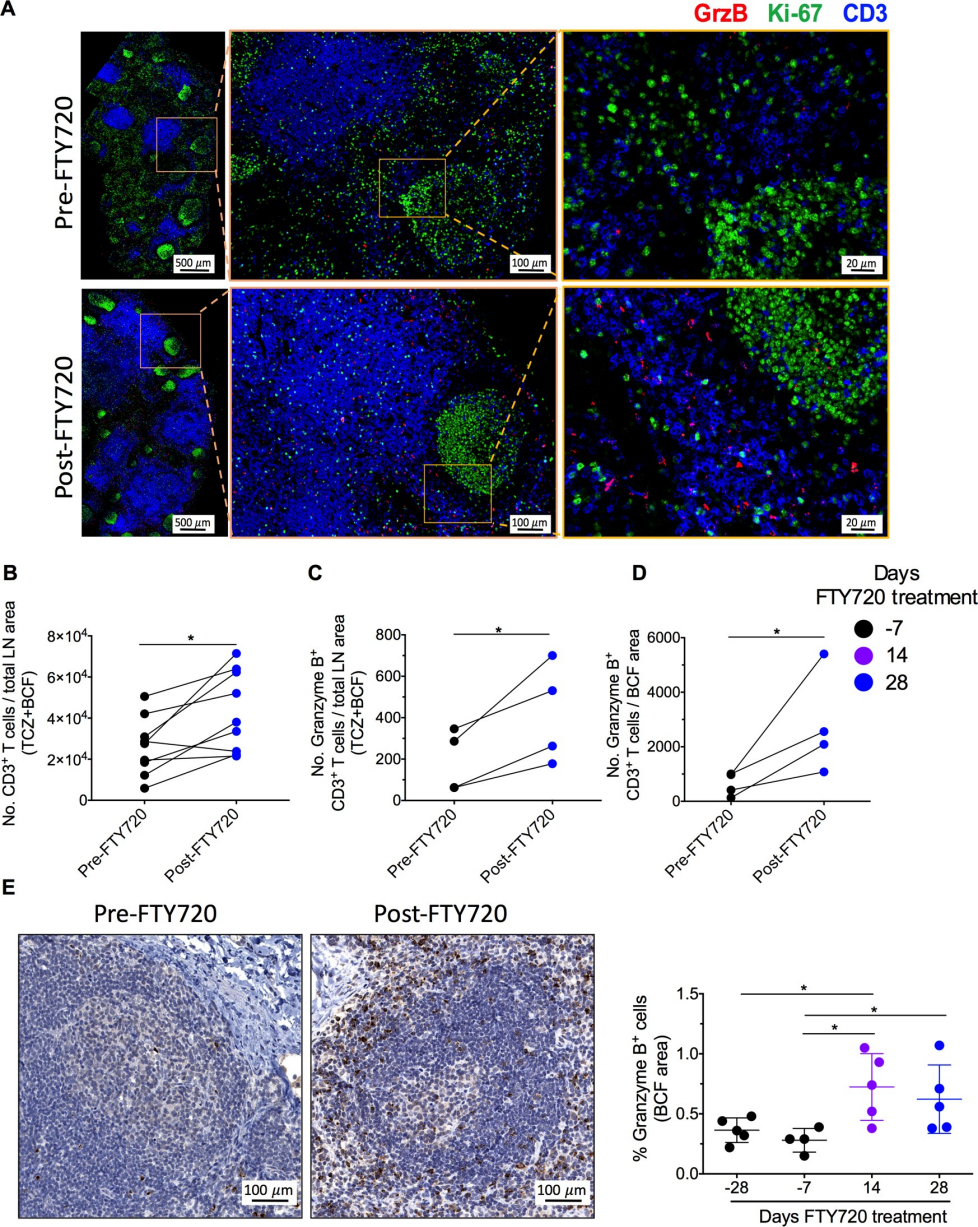
FTY720 accumulates T cells in LN. **(A)** Representative LN section stained with the indicated antibodies and imaged by Histo-Cytometry at pre-, and post-FTY720 treatment in a high dose group animal. Scale bars: left image = 500μm; middle image = 100μm; right image = 20μm **(B)** Absolute numbers of CD3+ (both high and low dose groups), **(C)** granzyme B+ CD3+ T cells per total LN area (T cell zone and B cell follicle; TCZ+ BCF), and **(D)** granzyme B+ CD3+ T cells per B cell follicle area (BCF) at pre-, and post-FTY720 treatment for high dose group. **(E)** Representative LN section stained with granzyme B and imaged by immunohistochemistry at pre-, and post-FTY720 treatment. Scale bar = 100μm (left panel). Frequency of granzyme B+ cells in B cell follicle area (BCF) at pre-, and post-FTY720 time points (right panel) for high dose group. In **(B)**, **(C)**, and (**D)** each dot indicate data from one animal. 3–5 different sections for each animal were examined, and one representative section was choosen for final analysis. In (**E**, right panel) data are presented as the mean ± SD. Statistical differences were assessed with a Mann-Whitney u-test in **(B), (C), (D)** or **(E)**. *P ≤ 0.05, **P ≤ 0.01, ***P ≤ 0.001, ****P ≤ 0.0001.

To provide an absolute quantification of LN immune cells, we performed histocytometry imaging for CD3 (use of formalin-fixed, paraffin embedded tissues preserves tissue architecture but precludes the use of available antibody clones reactive with RM CD8), granzyme B and Ki-67 in 9 of the 10 RMs that received FTY720. Staining for a representative RM before and at d 28 of FTY720 treatment is shown in **Fig 5A**. By combining confocal images with flow cytometry quantification, this technique allows a precise enumeration of a cell of interest. Consistently with the loss of T cells in circulation, FTY720 treated animals showed higher absolute numbers of CD3+ T cells in the LN area (**Fig 5B**; p = 0.01 for the two groups combined). In addition, most of the T cells following FTY720 treatment were localized in T cell zones in close proximity of the B cell follicles (BCF; **Fig 5A**). Consistent with the flow cytometric data of **Fig 4F**, FTY720 treatment increased the absolute number (**Fig 5C**) of CD3+ granzyme B+ T cells in 4 out of 4 RMs that received the highest dose (LN tissue was not available for this analysis in one animal of the high dose group) both in the total LN area (**Fig 5C**) and in the BCF (**Fig 5D**). To further define the immunologic impact of FTY720 on LN cytolytic T cells, we stained and quantified by immunohistochemistry (IHC) granzyme B+ T cells that localized in the BCF of the LN in all 5 RMs treated with the high dose of FTY720. Consistently with the absolute quantification, and as shown in the representative staining and in the graph of **Fig 5E**, the levels of follicular granzyme B+ T cells (quantified as % area of BCF) increased in every single animal following FTY720 treatment, with values significantly higher as compared to baseline at both day 14 and 28 of treatment (p = 0.031 and p = 0.04, respectively). Together, these data indicate that FTY720 treatment promotes retention of T cells, including those with a cytotoxic potential, in lymphoid sites where SIV persists, including BCF, during cART.

### FTY720 treatment limits the circulating reservoir and SIV infection of LN Tfh cells

**Fig 6:**
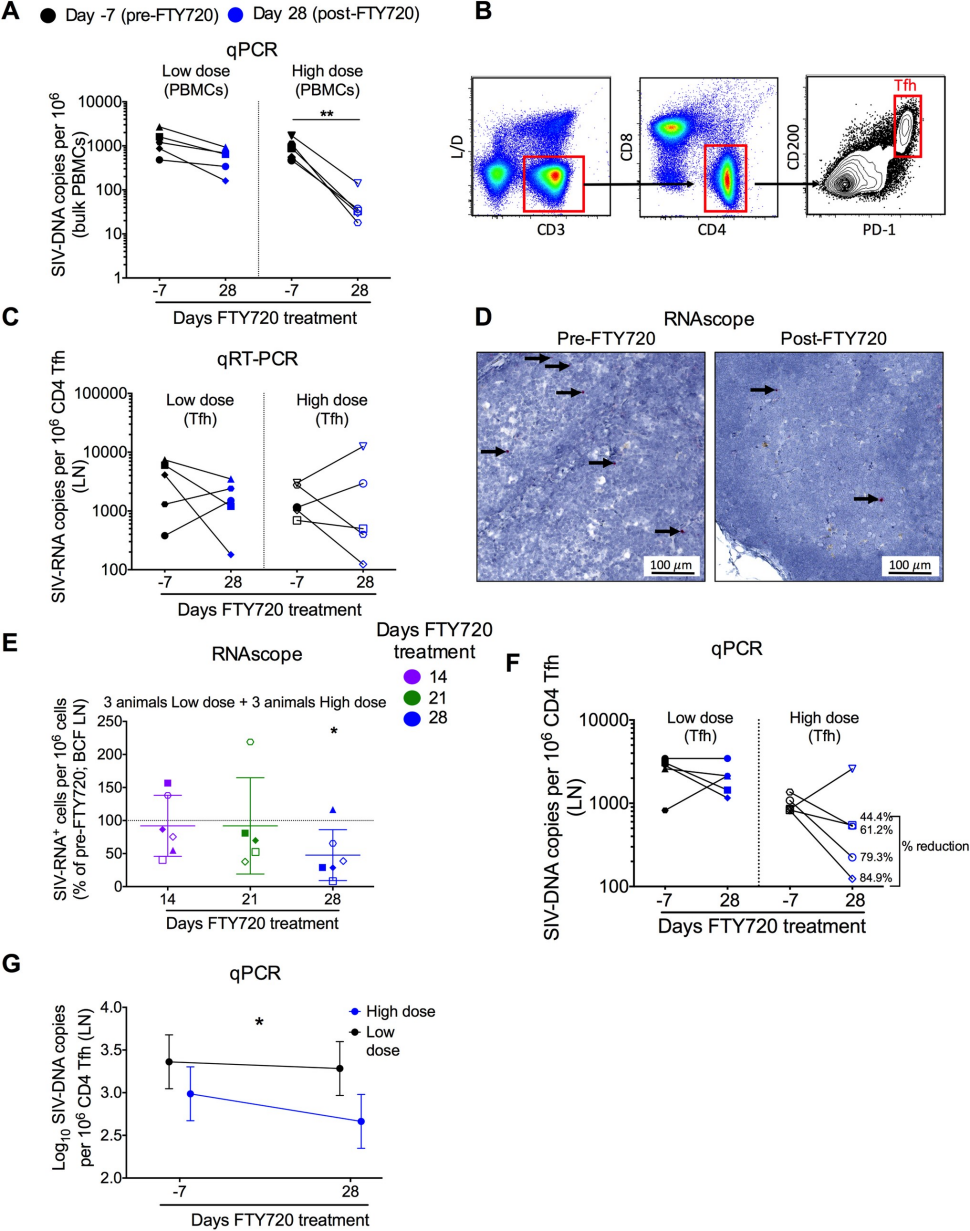
FTY720 treatment decreases SIV infection in blood and in LN Tfh cells. **(A)** Copies of total SIV_mac239_ DNA in bulk PBMCs quantified pre- and post-FTY720. **(B)** Representative sorting strategy of Tfh cells from lymph node (LN). **(C)** Copies of total SIV_mac239_ RNA per 10^6^ CD4 Tfh cells in LN quantified pre- and post-FTY720 treatment. **(D)** Representative LN section analyzed with RNAscope pre- and post-FTY720 treatment. Scale bar = 100μm. **(E)** Relative SIV-RNA+ cells per 10^6^ cells in the B-cell follicle analyzed with RNAscope post-FTY720 treatment for 3 animals from low dose group and 3 animals from high dose group. Values were normalized to the level of SIV-RNA+ cells per 10^6^ cells at baseline (pre-FTY720; set to 100%). **(F)** Copies of total SIV_mac239_ DNA per 10^6^ CD4 Tfh cells in LN quantified pre- and post-FTY720 treatment. **(G)** SIV-DNA regression analysis (ANCOVA) of pre- and post- FTY720 time points. Post-FTY720 treatment means were adjusted for pre-FTY720 differences. Each symbol represents individual animals. Averaged data are presented as the mean ± SD. Statistical differences were assessed with a one sample t-test or a Mann-Whitney u-test. ANCOVA analysis was performed in **(G)**. *P ≤ 0.05, **P ≤ 0.01, ***P ≤ 0.001, ****P ≤ 0.0001.

Since FTY720 was remarkably effective in reducing circulating CD4+ T cells, we postulated that this treatment would result in a reduction of the size of the SIV-reservoir in blood. To this aim, we determined the copies of SIV-DNA in circulating PBMCs isolated at baseline (d– 7) and at the end of FTY720 treatment (d 28) in all 10 treated RMs. Consistent with the extent of CD4+ T cell loss, animals treated with the highest dose of FTY720 showed a significant reduction in the SIV-DNA content as compared to baseline (p = 0.0079, **Fig 6A**). We then investigated if the lymph node sequestration of immune cells, including those with cytotoxic potential, was associated with any evidence of antiviral activity, including on Tfh cells that have been identified as important sites for HIV/SIV replication and persistence during cART [12, 13]. First, we sorted highly purified Tfh cells before (d– 7) and at d 28 after FTY720 treatment from the LN of all 10 cART-treated RMs included in the study. Tfh cells were defined as live, CD3+, CD4+, CD8-, PD-1hiCD200+ T cells; a representative gating strategy is shown in **Fig 6B**. The rationale for using CD200 instead of CXCR5 is that the former allow a better separation of Tfh cells. Of note, CD200 has been previously used to define Tfh in RMs [18], and the frequency of Tfh cells determined as PD-1+CD200+ or PD-1+CXCR5+ was virtually identical in our animals (**S6A** and **S6B Fig**). This approach allowed us to measure the SIV-DNA and SIV-RNA contents directly on purified Tfh cells and with a highly sensitive PCR method. Tfh SIV-RNA content was decreased at d 28 post FTY720 as compared to baseline in 6 out of 10 RMs, while increased in 4, with similar results for low dose group and high dose group 2 (**Fig 6C:** SIV-RNA copies per 10^6^ CD4 Tfh cells; **S6C Fig:** relative values, expressed as percentage of the SIV-RNA copies per 10^6^ CD4 Tfh cells at baseline, set to 100%). This finding was largely confirmed by “RNAscope” analysis (a stained section is shown in **Fig 6D**), showing that the number of SIV-RNA+ cells in B cell follicles progressively and significantly decreased during FTY720 treatment as compared to baseline in the 6 RMs (**Fig 6E**; 3 RMs from group 1 and 3 from group 2; p = 0.02). Furthermore, we found a measurable, selective (not present in other CD4 subsets, including central memory and effector memory, **S7 Fig**), and dose-dependent decrease in the frequency of Tfh cells harboring SIV-DNA. Indeed, Tfh cells in 4 out of 5 animals receiving the highest dose of FTY720 showed reduced SIV-DNA copies at d 28 of treatment as compared to baseline (**Fig 6F**). Although the overall difference for group 2 was only approaching significance (p = 0.14) due to the presence of an outlying animal with increased SIV-DNA content, there was an average 0.5 log_10_ reduction in Tfh SIV-DNA content between post and pre-FTY720 for the remaining four animals. Specifically, in those four animals FTY720 treatment induced a 44.4%, 61.2%, 79.3%, and 84.9% reduction in the Tfh cells SIV-DNA content as compared to their pre-FTY720 value. Consistent with a FTY720-dependent mechanism, the SIV-DNA content at d 28 of treatment was significantly lower in the five animals of high dose group as compared to the five animals of low dose group after ANCOVA model normalization for the pre-FTY720 SIV-DNA levels (**Fig 6G;** 0.65 log_10_ difference after normalization).

Altogether, these data suggest that SIP1 inhibition retains circulating immune cells including cytolytic effector cells in lymphoid tissues during cART. Even a short term (28 day) treatment can limit viral persistence during cART both in circulation as well as in lymphoid tissues in the majority of animals.

## Discussion

A gradient of S1P between lymph nodes and circulation mediates the egress of lymphocytes from lymph nodes promoting their entry into circulation. This study was designed to examine the tolerability and activity of the S1PR inhibitor FTY720 in the nonhuman primate model of SIV infection to explore the potential utility of this agent in retaining cytolytic antiviral lymphocytes in lymph nodes, sites of SIV persistence, from which they are typically excluded during cART [30]. We found that laboratory indices of tolerability and toxicity were unaffected by 4 weeks of FTY720 administration at doses up to 500 μg/kg per day; consistently, no adverse clinical events were noted by veterinary staff caring for these animals. FTY720 administration was associated with rapid profound decreases in the number of circulating T cells, including those with cytolytic potential. B lymphocyte numbers were also decreased. Earlier studies have suggested that the FTY720-dependent decrease in circulating T lymphocytes results from increased retention of lymphocyte populations in lymph nodes; however, it is very challenging to demonstrate that this is the case. Nonetheless, in this study, we showed for the first time in primates that the lymph nodes of FTY720 treated rhesus macaques contained greater numbers of CD3+ T cells, including those expressing granzyme B, perforin, and T-bet.

With administration of FTY720, there was a rapid increase in proportions of circulating CD4 and CD8 T cells in cell cycle (as measured by expression of Ki-67). Absolute numbers of circulating CD4+Ki-67+ T cells were, however, reduced. The proportions of cycling Ki-67+ CD4+ T cells were only modestly increased in lymphoid tissue during FTY720 administration. Thus, it is not clear whether this relative increase in cycling represents a homeostatic response to circulating lymphocytopenia, a relative exclusion of cycling cells from lymph node entry, a selective egress of these cells into circulation from tissues or perhaps a direct effect of FTY720 on T cell cycling. Since circulating CD4+Ki-67+ T cells express CCR7 at frequencies lower than those found in CD4+Ki-67- cell before FTY720 treatment, our data suggests that a lower ability of cycling cells to recirculate in lymphoid tissue contributes, at least in part, to the increased frequency of circulating CD4+Ki-67+ T cells observed following FTY720 treatment.

Lymph node germinal centers are the home of Tfh cells, a CD4+ T cell population that is enriched for sequences of HIV and SIV [12, 13]. The large majority of cytolytic T cells are typically excluded from these sites [22] and this exclusion is thought to represent a major barrier to the immunologic clearance of infected cells. In this work, it appears that FTY720 administration may have allowed some penetration of this barrier as T cell numbers were increased in lymph node, including those expressing granzyme B and localized in the BCF, and levels of proviral DNA were reduced in Tfh cells, but not in other lymph node CD4+ T cell populations, in the majority of treated animals. Whether this selectivity reflects the relatively greater transcriptional activity of SIV in Tfh [12] rendering them more “visible” to immune cells is plausible but not proven by this study. Furthermore, we cannot prove that the reduced infection of Tfh cells was the direct result of their increased killing from cytolytic T cells, or to address how FTY720 treatment impacted on CD8 T cell function [53]. The relatively selective antiviral effect against infected Tfh that is induced after FTY720 administration is also suggested by the results of RNAscope analysis that localized decreased SIV proviral RNA particularly to the germinal centers.

Due to the size of the study (5 animals in the high dose group) and the presence of one animal with an opposite readout, the ability of FTY720 to target Tfh cells harboring SIV-DNA needs to be investigated further in larger, controlled studies, and with methods that can enumerate Tfh cells harboring replication competent virus. Additional studies are also warranted to build on these findings with a design that may increase the potential utility of this strategy in targeting reservoirs of HIV/SIV persistence. In the current study, FTY720 was administered for a short period and after relatively prolonged cART-mediated viral suppression. If, as proposed, the antiviral effect is related to retention of cytolytic cells in lymphoid tissues, longer durations of FTY720 treatment could be more potent. Moreover, initiation of FTY720 administration at times when virus specific cytolytic cells are more frequent and/or functional, such for example, with initiation of cART could plausibly direct more cytolytic cells to sites of viral persistence. Also it is reasonable to design studies that include FTY720 as part of a combination regimen together with cytokines such as IL-15 or IL-2 that activate cytolytic cells, or with co-inhibitory receptor blockade that restores CD8 T cell function, or as part of an immunization strategy that generates and expands more antiviral cells with the intent of directing these cells to sites of HIV/SIV persistence. Of note, as a consequence of the very low number of circulating CD4+ T cells, FTY720-induced inhibition of T cell egress from LN resulted in a significant decrease of SIV-DNA and -RNA content in blood mononuclear cells. Thus, FTY720 administration has the potential to limit viral persistence in the critical cellular reservoir of Tfh cells while reducing the size of the viral reservoir in circulation.

FTY720 is approved as an effective treatment of multiple sclerosis. A proposed mechanism of its clinical activity is through retention of autoreactive immune cells in lymphoid tissues, however direct effects of this sphingosine 1 phosphate receptor blocker on neural cells also are implicated [35, 54]. Despite the induction of circulating lymphopenia in the setting of multiple sclerosis, the overall rate of infections was not increased by FTY720 administration, however, serious infections were seen in 2.3% of FTY720 treated patients and 1.6% of placebo treated patients and in the post-marketing setting, opportunistic infections have been reported (Gilenya, fingolimod). Thus if FTY720 is developed further as a strategy for the eradication of HIV, risk for infectious complications must be considered.

This study shows that modulation of the S1PR by FTY720 in cART-treated, SIV-infected RMs is tolerable and promotes retention of cytolytic T cells in lymphoid sites of SIV persistence. These data also demonstrate an impact on the circulating reservoir as well as on a critical cellular and anatomical reservoir of HIV persistence in LN. Collectively, these data provide rationale for testing FTY720, a drug approved for multiple sclerosis, in larger, controlled pre-clinical studies aimed at targeting HIV persistence in lymphoid tissues.

## Methods

### Animals, SIV-infection, antiretroviral therapy and FTY720 administration

We studied ten female Indian rhesus macaques (RMs; *Macaca mulatta*), all housed at the Yerkes National Primate Research Center, Atlanta, GA. All animals used in the present study were negative for known protective alleles in the rhesus macaque model of SIV/AIDS, Mamu-A*01, Mamu-B*08, and Mamu-B*17. All animals were infected intravenously with 300 TCID_50_ of SIV_mac239_ (provided by Chris Miller, UC Davis) (**Fig 1A**). Starting from day 42 post-infection all animals were treated for the entire duration of the study with a potent, combined antiretroviral regimen (cART) that included tenofovir (TDF; 5.1 mg/Kg per day), emtricitabine (FTC; 40 mg/Kg per day) and dolutegravir (DTG; 2.5 mg/Kg per day) formulated in a single daily injection (1ml/Kg per day; s.c.). cART was continued for approximately 7 months (up to day 258 p.i.). FTY720 was administered orally once a day for the last 28 days of cART treatment. The ten animals were divided in two groups of five: low dose group animals received a low dose (18 μg/Kg per day) while high dose group animals received a high dose (500 μg/Kg per day) of FTY720. FTY720 was started at 4 months of cART (day 162 p.i.) for the low dose group and at 6 months of cART for the high dose group (day p.i.). The rationale for waiting 6 months on cART for the high dose group was to have multiple time points when animals were aviremic; this design allowed us to confirm that differences were related to FTY720 treatment and not to additional 28-days of cART. At the end of the 28 days of FTY720 treatment, all animals underwent necropsy.

### Sample collection and processing

Collections of blood, lymph node (LN), and bone marrow (BM) aspirates were performed longitudinally during the entire study and at the necropsy. Blood samples were used for a complete blood count (CBC) and a comprehensive serum chemistry panel. Plasma was separated from EDTA-anticoagulated blood by centrifugation within 1 hour of phlebotomy. Density centrifugation was used to isolate peripheral blood mononuclear cells (PBMCs). For LN biopsies, the skin over the axillary or inguinal region was clipped and then surgically prepared. An incision was made in the skin over the LN, which was then exposed by blunt dissection and excised over clamps. Half of each LN biopsy was paraffin fixed for immunohistochemistry (IHC) or histo-cytometry analysis, while the other half was homogenized and passed through a 70-μm cell strainer to isolate lymphocytes. For BM aspirates, the area over the iliac crest was clipped and surgically prepared before aseptic introduction of a 14- to 20-gauge needle connected to a syringe (with or without heparin coating) into the bone. The desired volume was aspirated into the syringe. Suction was released before removing the BM needle. BM aspirations were performed from left and right iliac crest sides, and were limited to a volume of 1 to 1.5 ml/each to avoid contamination with blood. BM-derived cells were isolated by density gradient centrifugation. All samples were processed, stained, fixed (1% paraformaldehyde) and analyzed by flow cytometry within 24 hours of collection.

### Determination of plasma viral load RNA

SIV_mac239_ plasma viral load was quantified using a quantitative real-time PCR (qPCR) assay as described previously [55, 56].

### Flow cytometric analysis

Fourteen-parameter flow cytometric analysis was performed on PBMCs, and LN-derived cells according to standard procedures using a panel of monoclonal antibodies that we and others have shown to be cross-reactive with RM immune cells [13, 57]. Predetermined optimal concentrations of the following Abs were used: anti-CD3-APC-Cy7 (clone SP34-2), anti-Ki-67-Alexa Fluor 700 (clone B56), anti-CD95-APC (clone DX2), anti-CD95-PE-Cy5 (clone DX2), anti-CCR7-PE-Cy7 (clone 3D12), anti-CD28-PE-CF-594 (clone CD28.2), anti-CD21-PE (clone B-ly4), anti-CXCR3-Alexa Fluor 488 (clone 1C6/CXCR3), anti CD69-APC (clone FN50), anti-CD56-PE-Cy5 (clone B159), anti-CD14-PE-Cy7 (clone M5E2), anti-CD16-BV421 (clone 3G8), all from BD Biosciences; anti-CD4-BV605 (clone OKT4), anti-HLA-DR-BV570 (clone L243), anti-CD4-BV421 (clone OKT4), anti-CD20-PerCP-Cy5.5 (clone 2H7), anti-CD200-PE (clone OX104), anti-PD-1-BV421 (clone EH12.2H7), anti-CD4-BV650 (clone OKT4) all from Biolegend; anti-CXCR5-PerCP-eFluor710 (clone MU5UBEE), anti T-bet-PE (clone eBIO4B10) from eBioscience; anti-CD27-PE-Cy5 (clone 1A4LDG5), anti-NKG2a-APC (clone A60797) from Beckman Coulter; anti-CD8-Qdot705 (clone 3B5), anti-CD8-FITC (clone 3B5), anti-GrB-PE-Texas Red (clone GB11) and Aqua Live/Dead amine dye-AmCyan from Invitrogen; anti-CD38-FITC (clone AT-1) from STEMCELL Technologies; anti-Perforin-FITC (clone Pf-344) from MABTECH. Apoptotic cells were determined in frozen PBMCs by multicolor flow cytometry in CD3+, CD4+, and CD8+ T cells as percentage of cells reactive to Annexin V alone (early apoptosis) or Annexin V and 7-AAD (late apoptosis) following manufacturer instructions (PE Annexin V Apoptosis detection kit I, from BD Pharmingen). The antibodies used for this assay were: anti-CD3-APC-Cy7 (clone SP34-2) from BD Biosciences, anti-CD4-BV650 (clone OKT4) from Biolegend, and anti-CD8-FITC (clone 3B5) from Invitrogen. PBMCs incubated for 5 hours with 20μM camptothecin (Sigma-Aldrich) at 37°C were used as a positive control. Flow cytometric acquisition was performed on at least 100,000 CD3+ T cells on a BD LSRΙΙ Flow Cytometer driven by BD FACSDiva software. Analysis of the acquired data was performed by FlowJo software (Tree Star Inc.).

### FACS cell sorting

Mononuclear cells isolated from LN were stained with anti-CD3, anti-CD4, anti-CD8, anti-CD28, anti-CD95, anti-CCR7, anti-PD-1 and anti-CD200. Sorting of CD4+ Tfh (PD1+CD200+) was performed using a FACS AriaΙΙ (BD Biosciences) in samples collected before and during the FTY720 treatment. Post-sorting FACS analysis determined that sorted CD4+ T cell subsets were on average >96% pure.

### Confocal microscopy

Tissue imaging of formalin fixed paraffin embedded (FFPE) tissue sections was performed as described previously (*24*). Briefly, lymph nodes from FTY720 treated SIV infected monkeys were isolated and fixed in 10% formalin for 24hr at RT. Fixed tissues were embedded in paraffin. 5-micron tissue sections were cut by microtome (Leica Biosystems) and adhered to highly adhesive glass slides. Tissue sections were subjected to deparaffinization at 60°C for 30 min followed by antigen retrieval in Borg Decloaker RTU (Biocare Medical) at 110°C for 15 minutes. Tissue sections were then treated for permeabilization (0.3% Tritox-100 in PBS), stained with primary antibodies (O/N at 4°C), Secondary antibodies (2hr at RT), blocked with 10% goat serum (1hr at RT), and then incubated with conjugated antibodies (2hr at RT). Finally, slides were stained with JOJO-1 (Life Technologies) (20 min at RT) and mounted in Fluoromount-G (Southern Biotech). Stained slides were imaged on a NIKON (C2si) inverted confocal microscope equipped with 40X, 1.3 NA oil objective lens. Image acquisition was performed with NIS-elements software and analyzed in Imaris software version 8.2 (Bitplane). Spectral spillover between channels was corrected through live spectral un-mixing in NIS using data acquired from samples stained with single fluorochromes. Histo-cytometry analysis was performed as published earlier (*55*). Briefly, imaging datasets were segmented post-acquisition based on nuclear staining and average voxel intensities for all channels were extrapolated in Imaris. Channel statistics were exported to csv (comma separated values) files format and analyzed in FlowJo version 10.

The following antibodies were used for staining: Ki-67-BV421 (clone B56, BD Biosciences), Granzyme B (clone M7235, Dako antibody,), CD4-Alexa488 (polyclonal, R&D Systems), CD20-PB (eBioscience clone L26, conjugated in-house), anti-CD3 primary antibody (clone F7.2.38, Dako), Alexa680-conjugated anti-mouse IgG2a (Life technology), Alexa 546-conjugated anti-mouse IgG1 (Life technology) and Alexa 594 conjugated anti-mouse IgG1 secondary antibody (Thermo Fisher Scientific).

### Immunohistochemistry granzyme B staining

Immunohistochemical staining and quantification were performed as previously described [58]. In brief, immunohistochemistry on LN biopsies was performed on 5-μm tissue sections. Heat-induced epitope retrieval was performed by heating sections in 0.01% citraconic anhydride containing 0.05% Tween-20 then incubated with antibody to GzB (HPA003418, Sigma, 1:200) diluted in blocking buffer overnight 4°C. Slides were washed in 1× TBS with 0.05% Tween-20, endogenous peroxidases blocked using 1.5% (v/v) H_2_O_2_ in TBS, pH 7.4, for 5 min, incubated with rabbit or mouse Polink- 1 horseradish peroxidase (HRP) and developed with Immpact^TM^ DAB (3,3′-diaminobenzidine; Vector Laboratories) according to manufacturer’s recommendations. All slides were washed in H_2_O, counterstained with haematoxylin, mounted in Permount (Fisher Scientific), and scanned at high magnification (x200) using the ScanScope CS System (Aperio Technologies), yielding high-resolution data from the entire tissue section. Representative regions of interest (0.4mm^2^) were identified and high-resolution images extracted from these whole-tissue scans. The percentage area positive for GzB positive cells was quantified using Cell profiler version 3.1.5 [59].

### Next-generation RNAscope in situ hybridization and quantitative image analysis

We utilized a novel next-generation, ultrasensitive *in situ* hybridization technology for the detection of SIV RNA (RNAscope) with quantitative image analysis as previously described [60]. Animals have been selected based on the availability of a sufficient size of LN tissue and of SIV-RNA data by PCR. Regions of interest of 0.25 mm^2^ were selected within follicles and TCZ to maximize the size of tissue to be assessed. To obtain a better representation of the full LN we stained and quantified a total of 4 to 6 sections (5μm) per sample.

### Quantitation of cell-associated SIV-DNA and -RNA

Cell associated SIV *gag* DNA and RNA in sorted cells from LN were measured using quantitative PCR and RT PCR methods, essentially as described using high sensitivity assay formats [61].

### Statistical analysis

Data analyses were performed using GraphPad Prism (GraphPad Software, Inc., La Jolla, CA). The results are expressed as the mean ± SD. Statistical significance of immunophenotyping and viral data between time points and study groups were performed using a paired or Mann-Whitney unpaired u-test and ANOVA as appropriate. A P value less than 0.05 was considered statistically significant, and indicated as: *P ≤ 0.05, **P ≤ 0.01, ***P ≤ 0.001, ****P ≤ 0.0001.

### Ethics statement

All animal experimentations were conducted following guidelines established by the Animal Welfare Act and by the NIH’s Guide for the Care and Use of Laboratory Animals, 8^th^ edition. All procedures were performed in accordance with institutional regulations after review and approval by Emory University’s Institutional Animal Care and Usage Committee (IACUC; Permit number: 2002876) at Yerkes National Primate Research Center (YNPRC). Animal care facilities are accredited by the U.S. Department of Agriculture (USDA) and the Association for Assessment and Accreditation of Laboratory Animal Care (AAALAC) International. Appropriate procedures were performed to ensure that potential distress, pain, discomfort and/or injury was limited to that unavoidable in the conduct of the research plan. The sedative Ketamine (10 mg/kg) and/or Telazol (4 mg/kg) were applied as necessary for blood and tissue collections and analgesics were used when determined appropriate by veterinary medical staff. Euthanasia of RMs, using pentobarbital (100 mg/kg) under anesthesia, was performed at the end of the study by veterinary medical staff and according to IACUC endpoint guidelines. RMs were fed standard monkey chow (Jumbo Monkey Diet 5037, Purina Mills, St Louis, MO) twice daily, and half an orange per day. Consumption is monitored and adjustments are made as necessary depending on sex, age, and weight so that animals get enough food with minimum waste. SIV-infected RMs are singly caged but have visual, auditory, and olfactory contact with at least one social partner, permitting the expression of non-contact social behavior. The YNPRC enrichment plan employs several general categories of enrichment five times per week. Animals have access to more than one category of enrichment. IACUC proposals include a written scientific justification for any exclusions from some or all parts of the plan. Research-related exemptions are reviewed no less than annually. Clinically justified exemptions are reviewed more frequently by the attending veterinarian.

### Data and materials availability

All data supporting the findings of this study are presented in the article. Tenofovir (TDF) and emtricitabine (FTC) was obtained under a material transfer agreement (MTA) between Emory University and Gilead Sciences, Inc. Dolutegravir (DTG) was obtained under a material transfer agreement (MTA) between Emory University and ViiV Healthcare UK.

## Supporting information

S1 Fig FTY720 administration is well tolerated in cART-treated, SIV-infected RMs. **(A)** Serum chemistries and hematologic indices at baseline (d -7, pre-FTY720; black dots), and after FTY720 treatment (d 28, post-FTY720; blue dots) for low dose group and high dose group animals. **(B)** Weight at baseline (d -7, pre-FTY720; black dots), and after FTY720 treatment (d 28, post-FTY720; blue dots) for low dose group and high dose group of animals. Data are presented as the mean ± SD. Mann Whitney u-test was used to compare differences between pre-, and post-FTY720 time points within each group. *P ≤ 0.05, **P ≤ 0.01, ***P ≤ 0.001, ****P ≤ 0.0001.
(TIF)

S2 Fig FTY720 reduces circulating B and NK cell numbers. **A.** Representative staining of B (CD3-CD20+HLA-DR+) and NK (CD3-CD20-HLA-DR-NKG2A/C+CD8+) cells in blood. **(B)** Absolute numbers (cells/μl) of blood B cells and **(C)** NK cells at day -7 (pre-FTY720), and days 7, 14, 21, and 28 of FTY720 treatment for low dose group and high dose group. Data are presented as the mean ± SD. Statistical differences were assessed with a two-way ANOVA. *P ≤ 0.05, **P ≤ 0.01, ***P ≤ 0.001, ****P ≤ 0.0001.
(TIF)

S3 Fig FTY720 reduces levels of T cells and temporarily increases their expression of Ki-67 in BM. **(A)** Levels of bone marrow (BM) CD3+, **(B)** CD4+, and **(C)** CD8+ T cells, expressed as frequency of total lymphocytes, at day -7 (pre-FTY720), and days 14, 21, and 28 of FTY720 treatment for low dose group and high dose group. **(D)** Frequency of BM CD4+ and CD8+ T cells expressing Ki-67 at day -7 (pre-FTY720), and days 14, 21, and 28 of FTY720 treatment for (**D**) low dose group and **(E)** high dose group. Data are presented as the mean ± SD. Statistical differences were assessed with a two-way ANOVA. *P ≤ 0.05, **P ≤ 0.01, ***P ≤ 0.001, ****P ≤ 0.0001.
(TIF)

S4 Fig FTY720 reduces all circulating T cell subsets, including those producing cytotoxic molecules. **(A)** CD4+ (top panels), and CD8+ (bottom panels) Tcell subsets expressed in absolute numbers (cells/μl) at day -7 (pre-FTY720; black dots), and day 28 (post-FTY720; blue dots) for low dose group in blood (PBMCs). **(B)** Perforin, T-bet, and granzyme B expression on CD4+ (top panels), and CD8+ (bottom panels) T cells expressed in absolute numbers (cells/μl) at day -7 (pre-FTY720; black dots), and day 28 (post-FTY720; blue dots) for low dose group in blood (PBMCs). Data are presented as the mean ± SD. Statistical differences were assessed with a Mann-Whitney u-test. *P ≤ 0.05, **P ≤ 0.01, ***P ≤ 0.001, ****P ≤ 0.0001.
(TIF)

S5 Fig Frequency of lymphocyte populations in LN. **(A)** Frequency of CD4+ T cells, (**B)** CD8+ T cells, (**C)** NK cells, and (**D)** B cells at pre- and post-FTY720 treatment for low dose group and high dose group in LN. Data are presented as the mean ± SD. Statistical differences were assessed with a two-way ANOVA. *P ≤ 0.05, **P ≤ 0.01, ***P ≤ 0.001, ****P ≤ 0.0001.
(TIF)

S6 Fig Comparison of Tfh stainings in LN. Frequency of Tfh CD4+ Memory T cells at pre-, and post-FTY720 treatment defined by CXCR5+PD-1+ (black dots) or CD200+PD-1+ (orange dots) in LN for **(A)** low dose group, and **(B)** high dose group. **(C)** Relative copies of total SIV_mac239_ RNA per 10^6^ CD4 Tfh cells in LN quantified at post-FTY720 treatment. Values were normalized to copies of total SIV_mac239_ RNA per 10^6^ CD4 Tfh cells at baseline (pre-FTY720; set to 100%). Data are presented as the mean ± SD. Statistical differences were assessed with a Mann-Whitney u-test.
(TIF)

S7 Fig SIV infection in central and effector memory CD4+ T cells in LN. **(A), (B)** Copies of total SIV_mac239_ DNA and **(C), (D)** SIV_mac239_ RNA per 10^6^ central memory (CM, **A, C**), and effector memory (EM, **B, D**) CD4+ T cells in LN quantified pre- and post-FTY720 treatment. Statistical differences were assessed with a Mann-Whitney u-test.
(TIF)

S1 Table Plasma viral loads. Longitudinal plasma SIV_mac239_ RNA levels expressed as copies/ml (LOD, 60 copies/ml) are shown for each individual animal from low dose group (top table) and high dose group (bottom table). Viral loads below LOD are indicated as 30 copies/ml.
(TIF)

S2 Table Toxicity and tolerability measurements. Serum chemistries indices at baseline (pre-FTY720) and day 28 of FTY720 treatment (post-FTY720) from low dose group (top table) and high dose group (bottom table).
(TIF)
