# Supplementary figures and images for: Fingolimod retains cytolytic T cells and limits T follicular helper cell infection in lymphoid sites of SIV persistence
Source: PLoS Pathog. 2019 Oct 18;15(10):e1008081. doi: 10.1371/journal.ppat.1008081 (PMC6834281; doi:10.1371/journal.ppat.1008081)

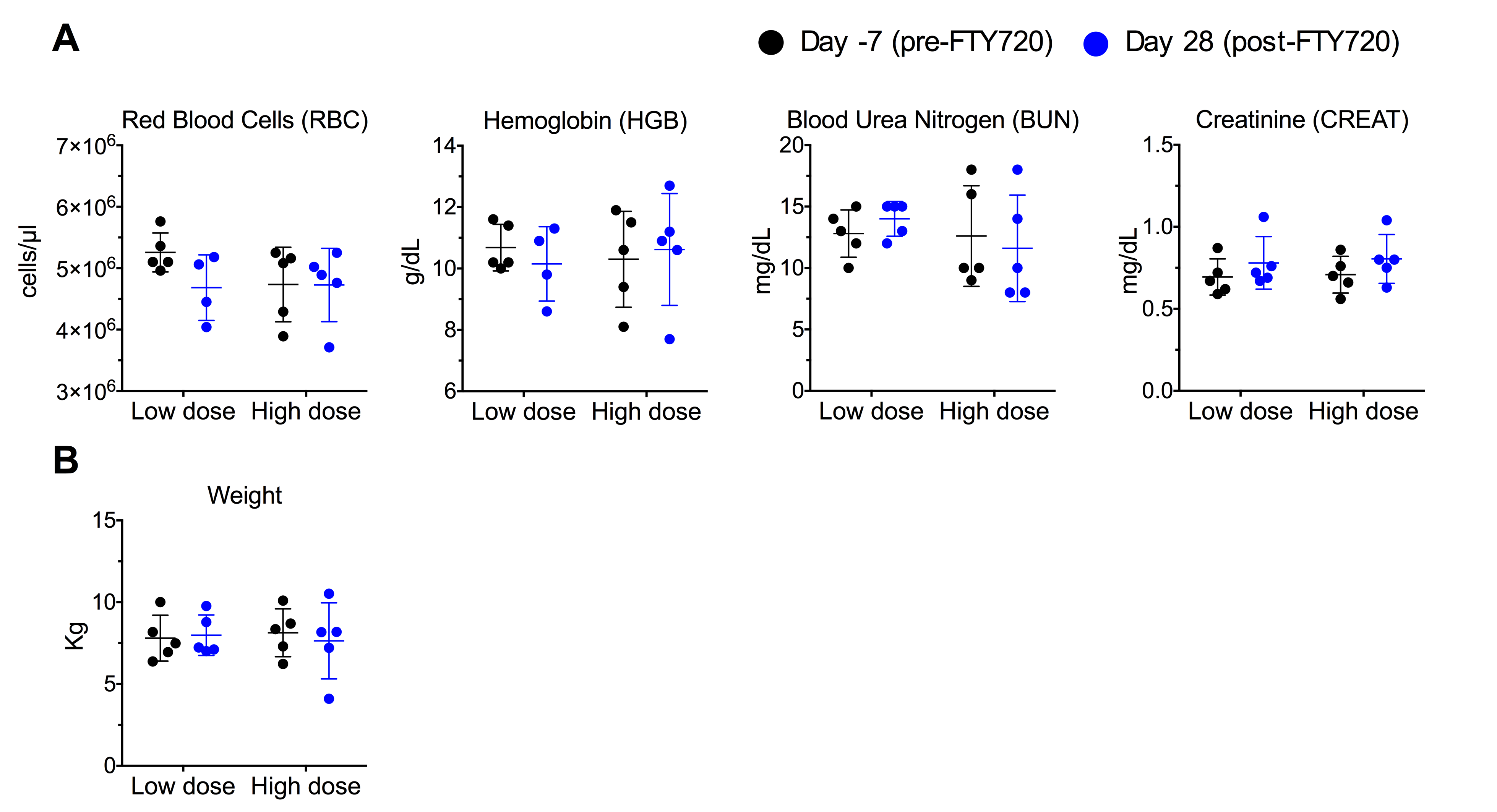

Supplement: S1 Fig — (A) Serum chemistries and hematologic indices at baseline (d -7, pre-FTY720; black dots), and after FTY720 treatment (d 28, post-FTY720; blue dots) for low dose group and high dose group animals. (B) Weight at baseline (d -7, pre-FTY720; black dots), and after FTY720 treatment (d 28, post-FTY720; blue dots) for low dose group and high dose group of animals. Data are presented as the mean ± SD. Mann Whitney u-test was used to compare differences between pre-, and post-FTY720 time points within each group. *P ≤ 0.05, **P ≤ 0.01, ***P ≤ 0.001, ****P ≤ 0.0001. (TIF) [file ppat.1008081.s001.tif]

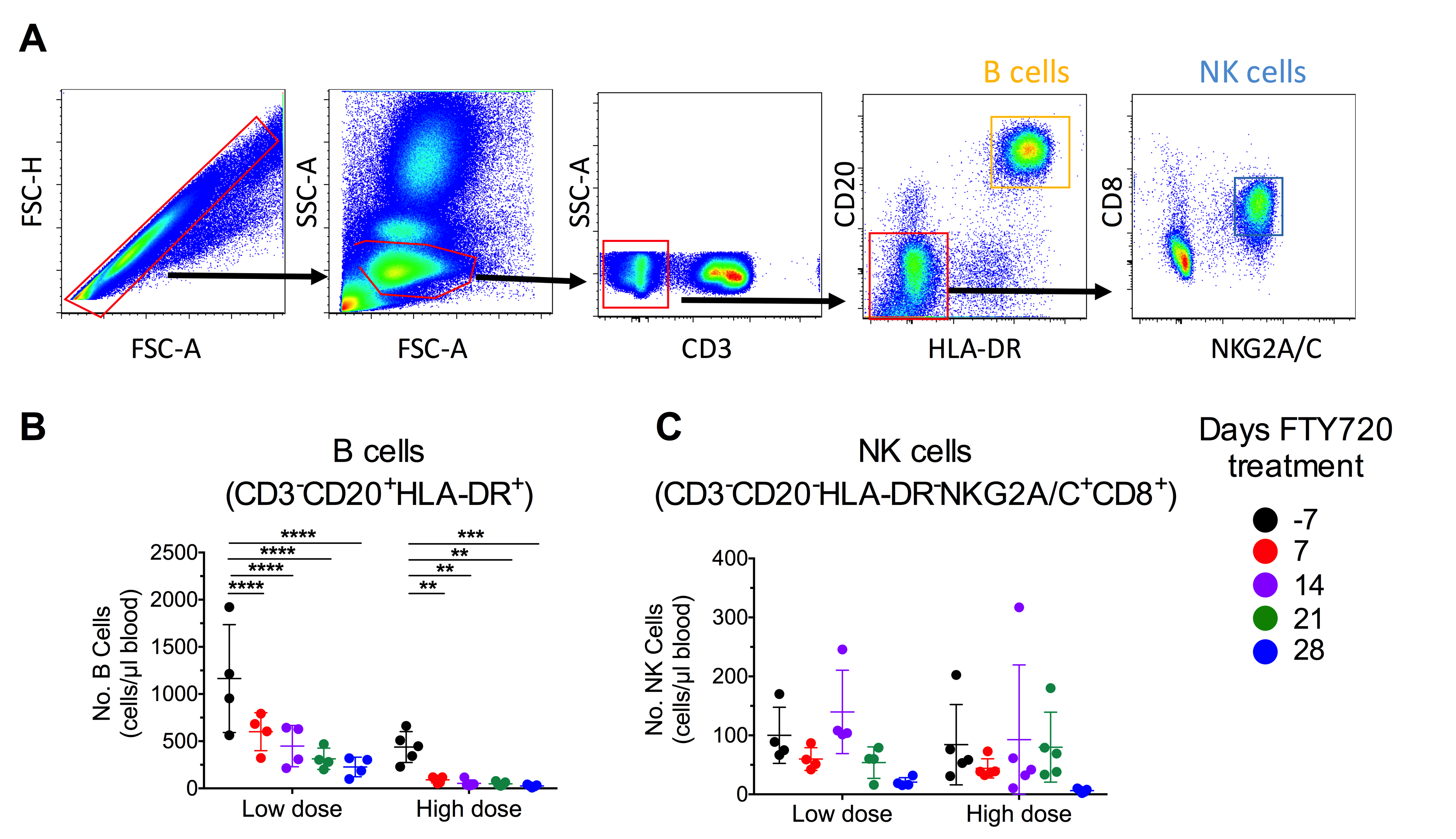

Supplement: S2 Fig — A. Representative staining of B (CD3-CD20+HLA-DR+) and NK (CD3-CD20-HLA-DR-NKG2A/C+CD8+) cells in blood. (B) Absolute numbers (cells/μl) of blood B cells and (C) NK cells at day -7 (pre-FTY720), and days 7, 14, 21, and 28 of FTY720 treatment for low dose group and high dose group. Data are presented as the mean ± SD. Statistical differences were assessed with a two-way ANOVA. *P ≤ 0.05, **P ≤ 0.01, ***P ≤ 0.001, ****P ≤ 0.0001. (TIF) [file ppat.1008081.s002.tif]

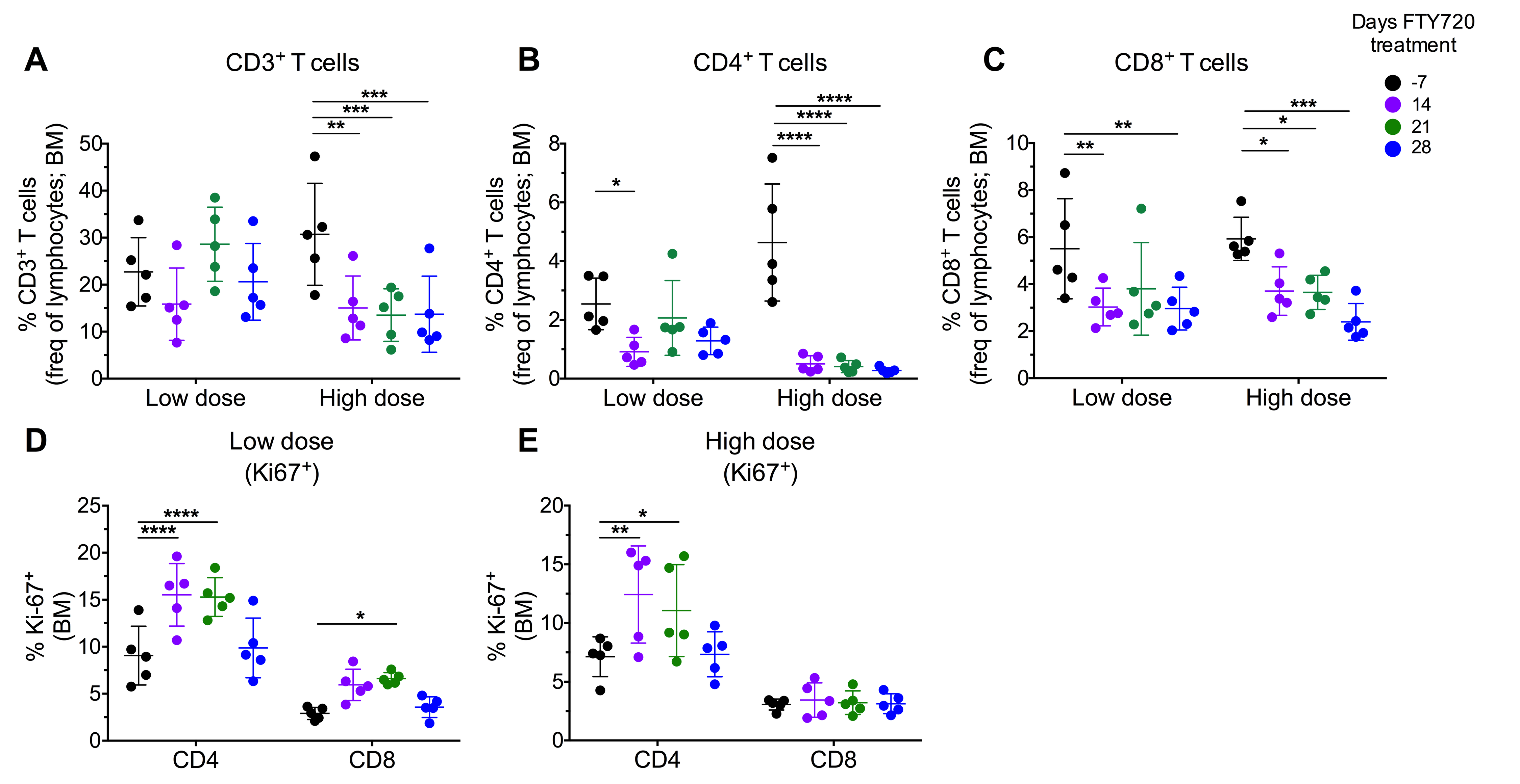

Supplement: S3 Fig — (A) Levels of bone marrow (BM) CD3+, (B) CD4+, and (C) CD8+ T cells, expressed as frequency of total lymphocytes, at day -7 (pre-FTY720), and days 14, 21, and 28 of FTY720 treatment for low dose group and high dose group. (D) Frequency of BM CD4+ and CD8+ T cells expressing Ki-67 at day -7 (pre-FTY720), and days 14, 21, and 28 of FTY720 treatment for (D) low dose group and (E) high dose group. Data are presented as the mean ± SD. Statistical differences were assessed with a two-way ANOVA. *P ≤ 0.05, **P ≤ 0.01, ***P ≤ 0.001, ****P ≤ 0.0001. (TIF) [file ppat.1008081.s003.tif]

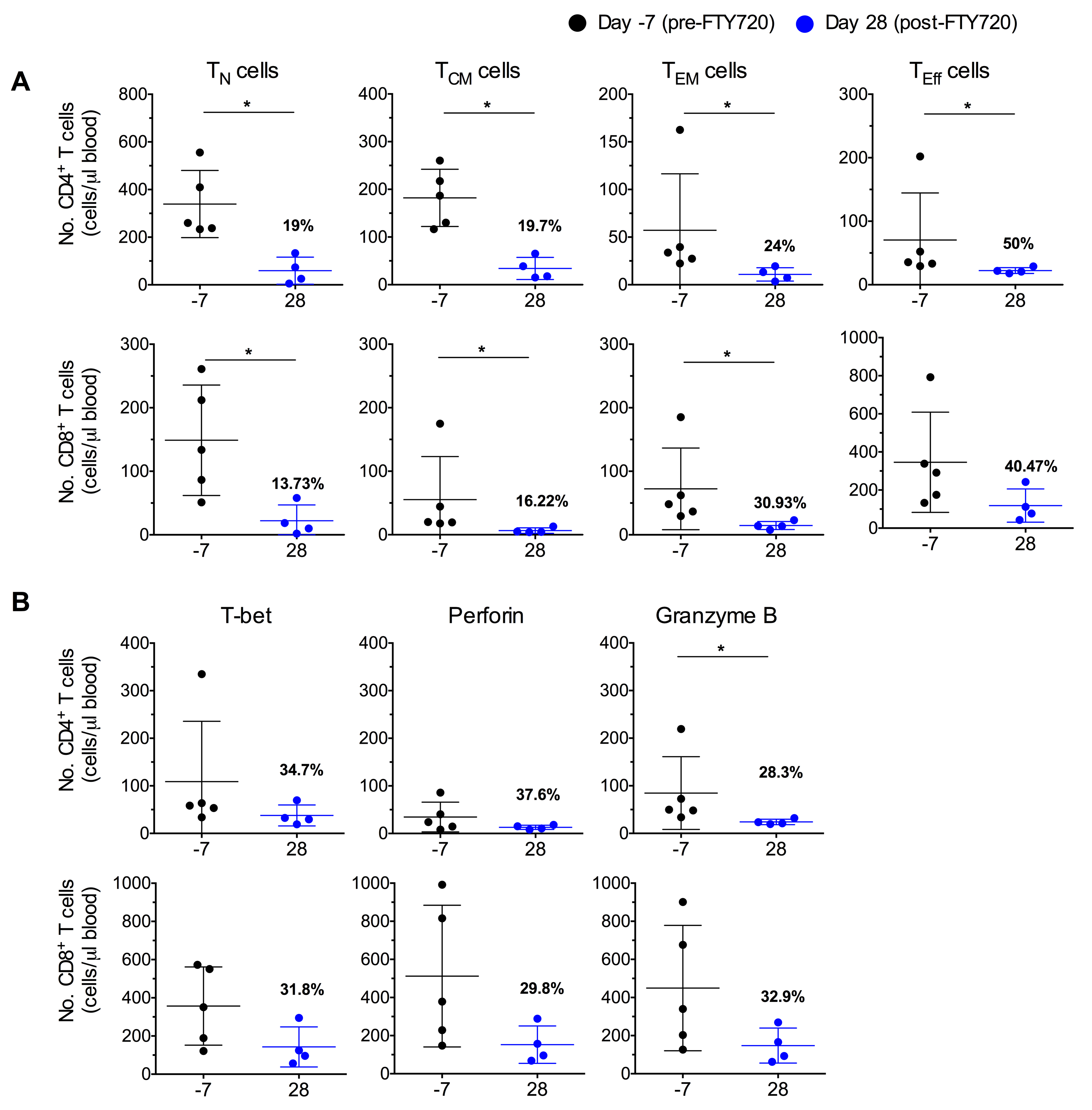

Supplement: S4 Fig — (A) CD4+ (top panels), and CD8+ (bottom panels) Tcell subsets expressed in absolute numbers (cells/μl) at day -7 (pre-FTY720; black dots), and day 28 (post-FTY720; blue dots) for low dose group in blood (PBMCs). (B) Perforin, T-bet, and granzyme B expression on CD4+ (top panels), and CD8+ (bottom panels) T cells expressed in absolute numbers (cells/μl) at day -7 (pre-FTY720; black dots), and day 28 (post-FTY720; blue dots) for low dose group in blood (PBMCs). Data are presented as the mean ± SD. Statistical differences were assessed with a Mann-Whitney u-test. *P ≤ 0.05, **P ≤ 0.01, ***P ≤ 0.001, ****P ≤ 0.0001. (TIF) [file ppat.1008081.s004.tif]

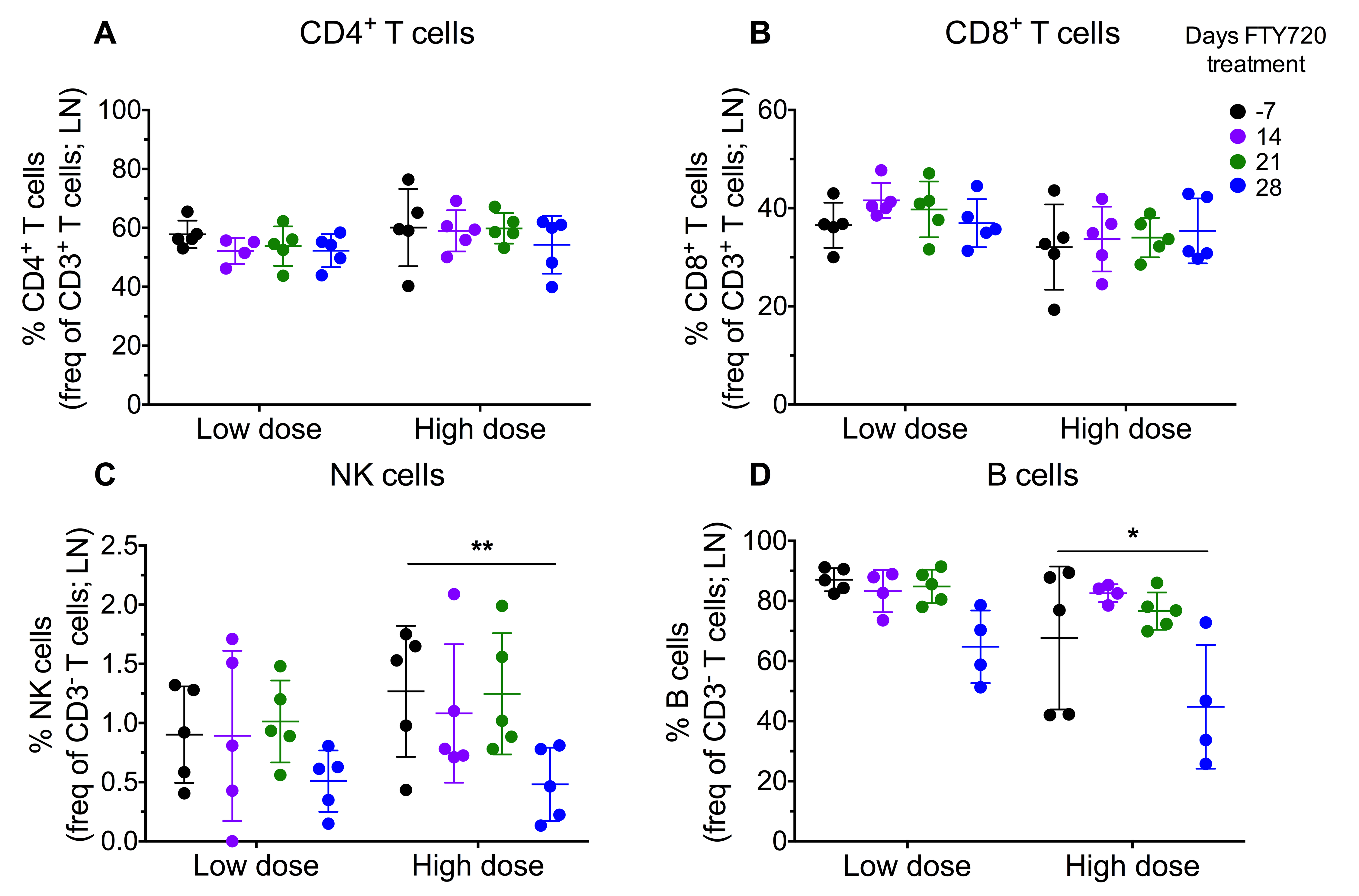

Supplement: S5 Fig — (A) Frequency of CD4+ T cells, (B) CD8+ T cells, (C) NK cells, and (D) B cells at pre- and post-FTY720 treatment for low dose group and high dose group in LN. Data are presented as the mean ± SD. Statistical differences were assessed with a two-way ANOVA. *P ≤ 0.05, **P ≤ 0.01, ***P ≤ 0.001, ****P ≤ 0.0001. (TIF) [file ppat.1008081.s005.tif]

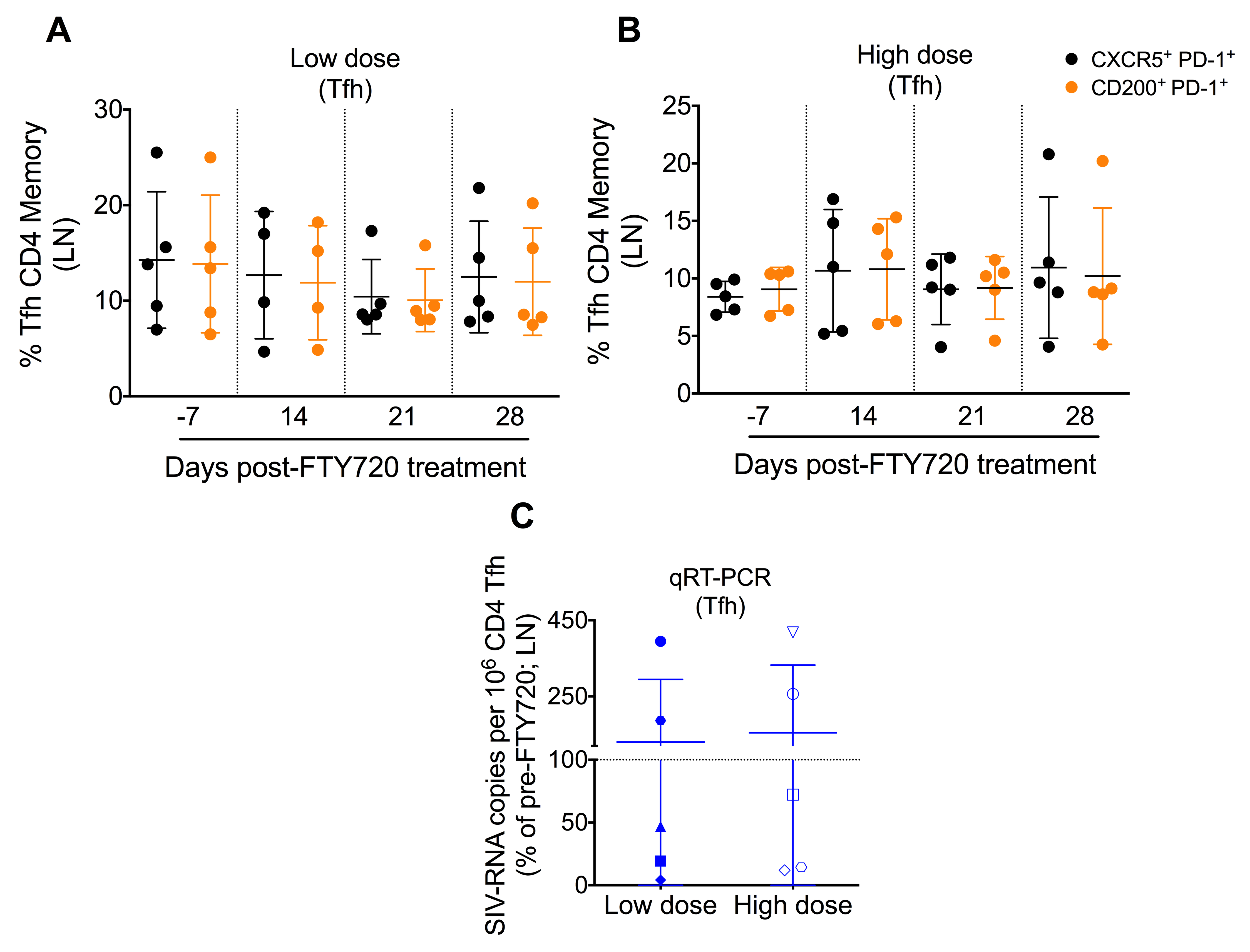

Supplement: S6 Fig — Frequency of Tfh CD4+ Memory T cells at pre-, and post-FTY720 treatment defined by CXCR5+PD-1+ (black dots) or CD200+PD-1+ (orange dots) in LN for (A) low dose group, and (B) high dose group. (C) Relative copies of total SIVmac239 RNA per 106 CD4 Tfh cells in LN quantified at post-FTY720 treatment. Values were normalized to copies of total SIVmac239 RNA per 106 CD4 Tfh cells at baseline (pre-FTY720; set to 100%). Data are presented as the mean ± SD. Statistical differences were assessed with a Mann-Whitney u-test. (TIF) [file ppat.1008081.s006.tif]

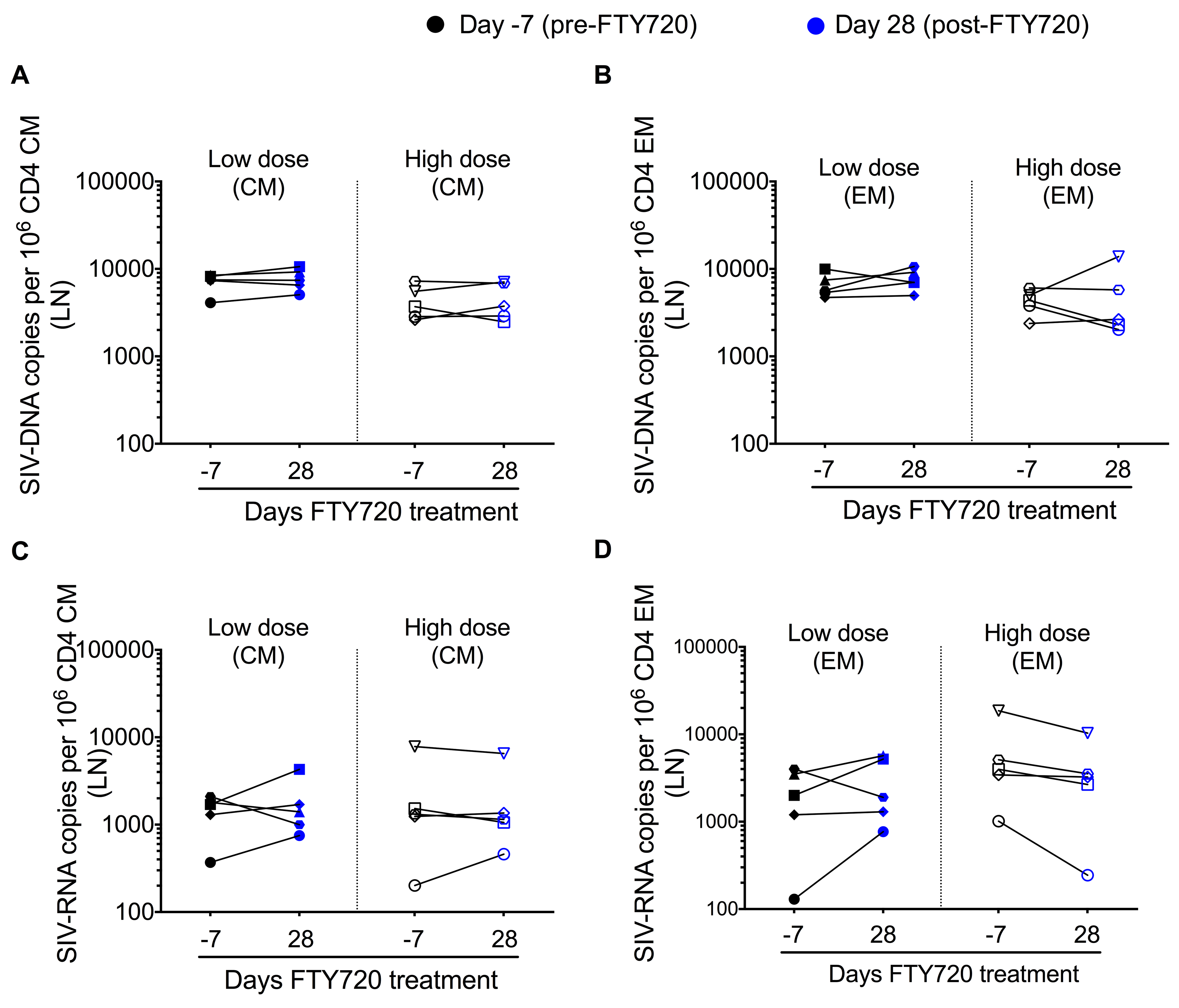

Supplement: S7 Fig — (A), (B) Copies of total SIVmac239 DNA and (C), (D) SIVmac239 RNA per 106 central memory (CM, A, C), and effector memory (EM, B, D) CD4+ T cells in LN quantified pre- and post-FTY720 treatment. Statistical differences were assessed with a Mann-Whitney u-test. (TIF) [file ppat.1008081.s007.tif]

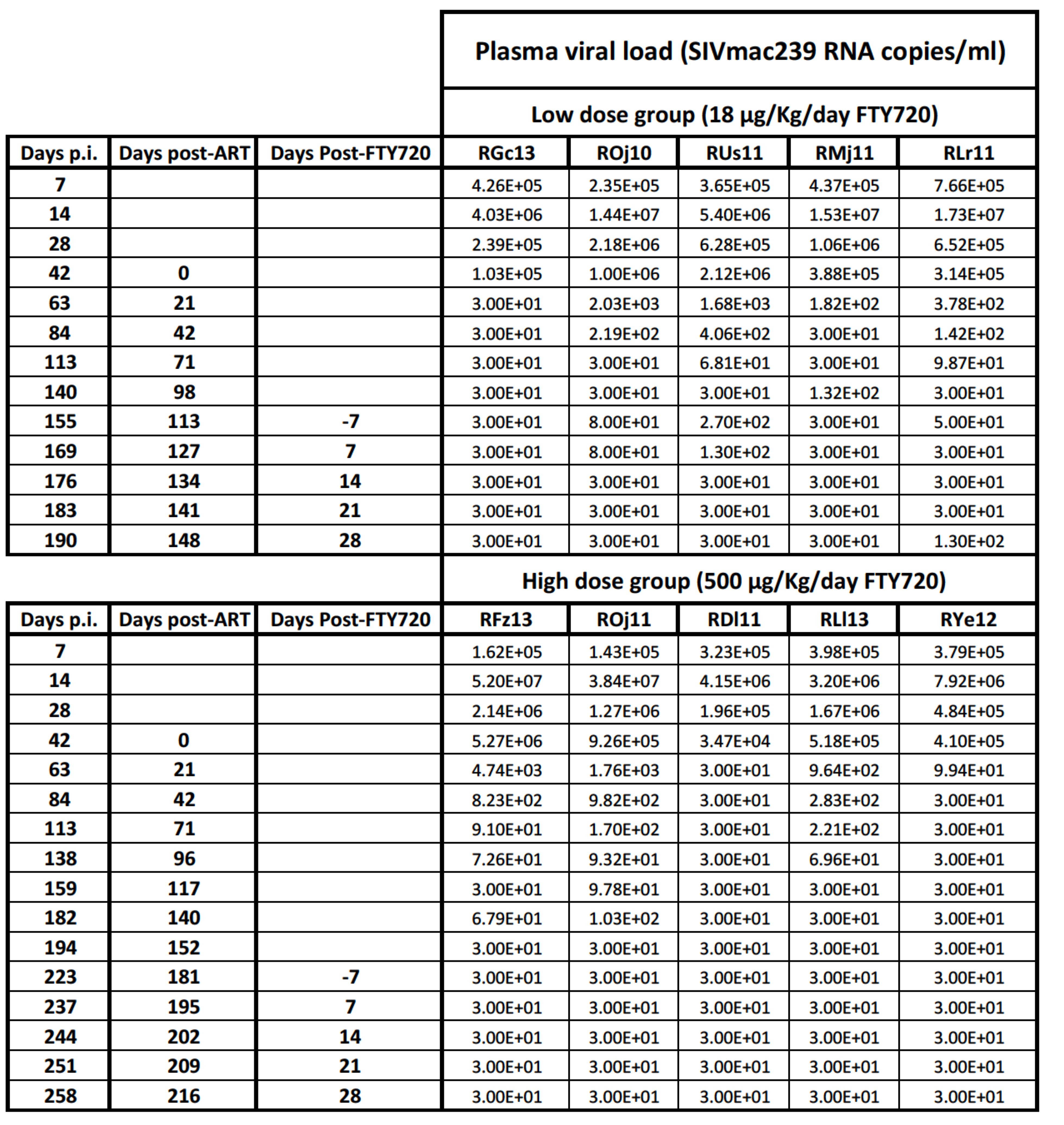

Supplement: S1 Table — Longitudinal plasma SIVmac239 RNA levels expressed as copies/ml (LOD, 60 copies/ml) are shown for each individual animal from low dose group (top table) and high dose group (bottom table). Viral loads below LOD are indicated as 30 copies/ml. (TIF) [file ppat.1008081.s008.tif]

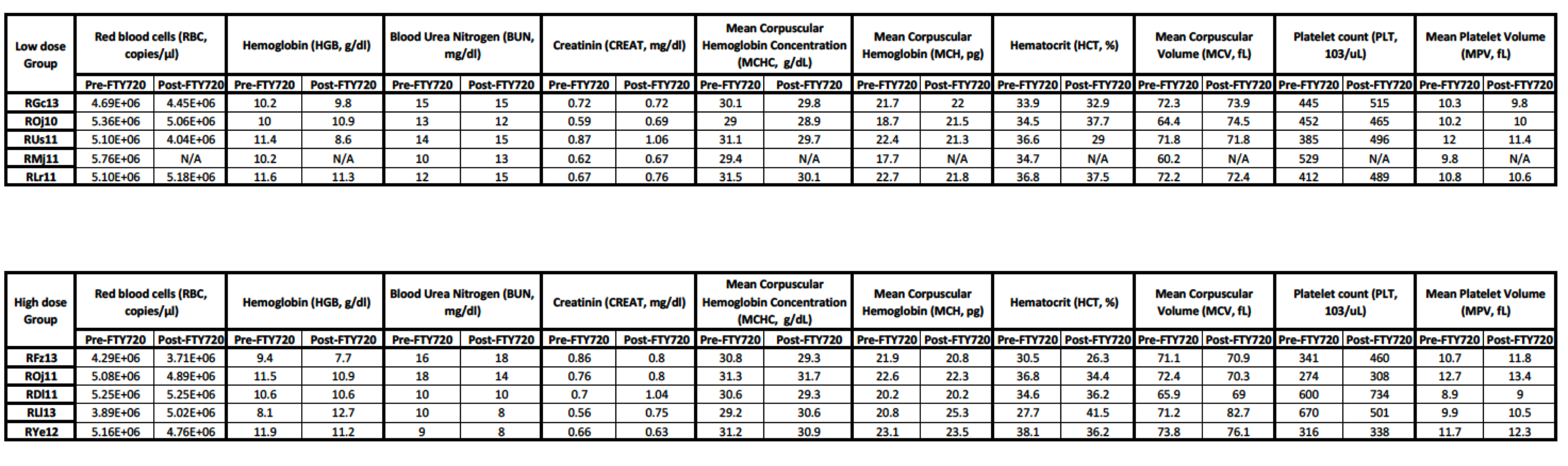

Supplement: S2 Table — Serum chemistries indices at baseline (pre-FTY720) and day 28 of FTY720 treatment (post-FTY720) from low dose group (top table) and high dose group (bottom table). (TIF) [file ppat.1008081.s009.tif]
